# Thalidomide targets EGFL6 to inhibit EGFL6/PAX6 axis-driven angiogenesis in small bowel vascular malformation

**DOI:** 10.1007/s00018-020-03465-3

**Published:** 2020-02-01

**Authors:** Chao-Tao Tang, Qing-Wei Zhang, Shan Wu, Ming-Yu Tang, Qian Liang, Xiao-Lu Lin, Yun-Jie Gao, Zhi-Zheng Ge

**Affiliations:** 1 Division of Gastroenterology and Hepatology, Key Laboratory of Gastroenterology and Hepatology, Ministry of Health, Renji Hospital, School of Medicine, Shanghai Jiao Tong University, Shanghai Institute of Digestive Disease, Shanghai, 200001 China; 2 Department of Digestive Endoscopy, Provincial Clinic Medical College, Fujian Medical University, Fujian Provincial Hospital, Fuzhou, 350001 China

**Keywords:** Angiodysplasia, Obscure gastrointestinal bleeding, Proteasome-dependent degradation

## Abstract

**Background:**

Small bowel vascular malformation disease (SBVM) is the most common cause of obscure gastrointestinal bleeding (OGIB). Several studies suggested that EGFL6 was able to promote the growth of tumor endothelial cells by forming tumor vessels. To date, it remains unclear how EGFL6 promotes pathological angiogenesis in SBVM and whether EGFL6 is a target of thalidomide.

**Methods:**

We took advantage of SBVM plasma and tissue samples and compared the expression of EGFL6 between SBVM patients and healthy people via ELISA and Immunohistochemistry. We elucidated the underlying function of EGFL6 in SBVM in vitro and by generating a zebrafish model that overexpresses EGFL6, The cycloheximide (CHX)-chase experiment and CoIP assays were conducted to demonstrate that thalidomide can promote the degradation of EGFL6 by targeting CRBN.

**Results:**

The analysis of SBVM plasma and tissue samples revealed that EGFL6 was overexpressed in the patients compared to healthy people. Using in vitro and in vivo assays, we demonstrated that an EMT pathway triggered by the EGFL6/PAX6 axis is involved in the pathogenesis of SBVM. Furthermore, through in vitro and in vivo assays, we elucidated that thalidomide can function as anti-angiogenesis medicine through the regulation of EGFL6 in a proteasome-dependent manner. Finally, we found that CRBN can mediate the effect of thalidomide on EGFL6 expression and that the CRBN protein interacts with EGFL6 via a Lon N-terminal peptide.

**Conclusion:**

Our findings revealed a key role for EGFL6 in SBVM pathogenesis and provided a mechanism explaining why thalidomide can cure small bowel bleeding resulting from SBVM.

**Electronic supplementary material:**

The online version of this article (10.1007/s00018-020-03465-3) contains supplementary material, which is available to authorized users.

## Introduction

Small bowel vascular malformation (SBVM) accounts for 30–40% of cases of small bowel bleeding (also called obscure gastrointestinal bleeding, OGIB), which is the most common cause of OGIB in people older than 40 years of age [1]. A 13-year follow-up study showed that 16–64% of cases would lead to iron deficiency anemia or require a blood transfusion, which undoubtedly is a major burden [2]. Histologically, SBVM is characterized by dilated communication between veins and capillaries, and usually, these vessels are thin and immature, without a smooth muscle layer [1]. It has been suggested that the pathophysiology of SBVM involves chronical hypoxia and the aging process, whereas others implicate a number of factors such as HIF [3], VEGF, Tie2, and angiopoietin 1 and 2 [4]. However, the underlying mechanism involved in the pathogenesis of SBVM remains unknown. Moreover, although our team first demonstrated that thalidomide is an effective therapy for SBVM patients [5], its specific targets remain unclear.

Angiogenesis involves vessel sprouting in which subpopulations of endothelial cells acquire mesenchymal phenotypes to promote their ability to proliferate and migrate [6]. In this process, endothelial cells are stimulated by many factors such as VEGF-A, Delta-like 4 (DLL4), and Notch1 [4, 7]. EGFL6, a member of EGF repeat superfamily, was found to be expressed during early development, especially in fetal tissues (lung, umbilical cord, and placenta), whereas in normal adult tissues, its expression levels are low or absent [8]. Moreover, the structure of EGFL6 protein is more similar to Notch than EGF, which suggests that EGFL6 is functionally related to the Notch interactome [8]. To date, EGFL6 has been reported to be upregulated in many tumors such as breast, lung, and nasopharyngeal cancer [9, 10] and participates in the tumor progression and metastasis. For instance, EGFL6 was recently reported to accelerate the growth and metastasis of nasopharyngeal carcinoma by regulating the expression of the Akt pathway [9]. EGFL6 promotes the invasive phenotype of breast cancer by inducing the epithelial–mesenchymal transition (EMT) [10]. This protein has multiple roles in promoting carcinogenesis, and the most important function of EGFL6 is linked to the regulation of angiogenesis.

The angiogenic function of EGFL6 was first implicated in hepatocellular carcinoma [11]. Since then, EGFL6 was reported to be overexpressed in tumors and in abnormal microenvironments such as the bone local environment and ovarian cancer [12, 13]. Recently, EGFL6 was found to be significantly upregulated in tumor tissues versus wound tissues or normal endothelial cells, which in turn enhances tumor angiogenesis by mediating Tie2/PI3K/AKT signaling in hypoxic environments, thereby suggesting that the targeting of EGFL6 could be an effective treatment target [14]. Moreover, although EGFL6 expression has been shown to be correlated with tumorigenesis, angiogenesis, and vasculogenesis, its unique role in SBVM remains unclear.

Thalidomide was synthesized and used as a tranquilizer in 1954 and was discontinued after it was found to have fatal teratogenic side effects. In early studies on the teratogenic mechanism of thalidomide, it was reported that the drug alters the activity of an E3 ligase by binding to one of its targets, CRBN protein (synthesizing E3 ubiquitinated ligase complex), which then affects the interaction between another target and the E3 ligase. Subsequently, for treatment of myeloma, it was found that thalidomide activates the ubiquitination protease system (UPS) by binding to CRBN, and that transcription factors IKZF1 and IKZF3 are substrates of this pathway [15]. In recent years, it has been found that thalidomide imparts an anti-angiogenic effect by inhibiting the expression of VEGF and basic fibroblast growth factor (bFGF) [16]. However, the specific mechanism of thalidomide in the treatment of small intestinal vascular malformations remains unclear.

Here, we first analyzed the level of EGFL6 expression in the serum of SBVM patients and sample tissues by ELISA and Immunohistochemistry (IHC). Using in vitro and in vivo assays, we elucidated the mechanism by which EGFL6 promotes angiogenesis. More importantly, we measured EGFL6 expression in the blood of SBVM patients who were treated with thalidomide compared to baseline levels (before thalidomide treatment). In vitro assay, we found that thalidomide promoted the degradation of EGFL6 by targeting the CRBN protein.

## Materials and methods

Note: The full Materials and Methods section is included in the supplementary material 1.

## Results

### EGFL6 is overexpressed in SBVM patients and is associated with bleeding

**Fig. 1:**
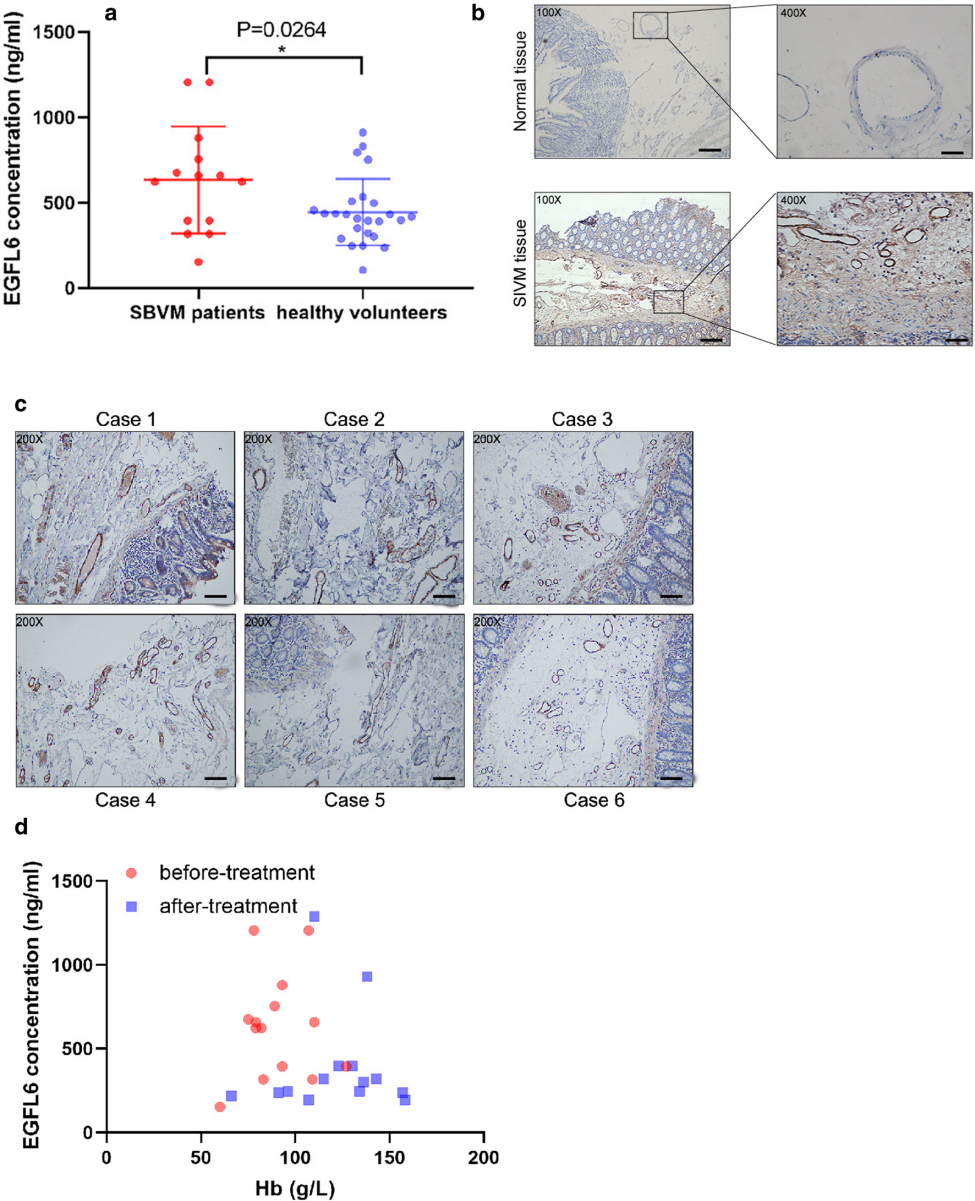
EGFL6 expression is upregulated in SBVM tissues and serum of patients and correlates with the level of Hb. **a** ELISA was used to determine the level of EGFL6 in plasma between healthy volunteers and SBVM patients. **b** Representative images of IHC staining of EGFL6 in SBVM and normal tissues. Scale bars: left, 200 µm; right, 50 µm. **c** EGFL6 protein expressions in six SBVM samples were analyzed by IHC. Scale bars: 100 µm. **d** Correlation between EGFL6 expression and hemoglobin (Hb) level was analyzed by Graphpad software

To investigate the expression of EGFL6 in SBVM patients and healthy volunteers, we recruited 14 SBVM patients diagnosed by capsule endoscopy and 25 healthy volunteers to perform an ELISA with blood plasma. As expected, the results showed that the level of EGFL6 expression was higher in SBVM patients compared with healthy volunteers (*P* < 0.05) (Fig. 1a). Additionally, immunohistochemistry inspection of the collected surgical specimen of SBVM revealed that the EGFL6 protein was upregulated in SBVM compared to the expression in normal tissues (Fig. 1b). Although only a few specimens were analyzed, we found that EGFL6 was overexpressed in abnormal vessels, indicating that EGFL6 promotes excessive angiogenesis in SBVM (Fig. 1c). To evaluate the correlation between the expression of EGFL6 and Hb before and after treatment, we generated a scatter plot. We found that the level of EGFL6 was high, and the level of Hb was low in patients before treatment, whereas after treatment, the level of EGFL6 decreased and the level of Hb increased (Fig. 1d). The increase in EGFL6 expression in the SBVM samples suggests that EGFL6 may be associated with the incidents of bleeding and development of SBVM.

### EGFL6 promotes angiogenesis in vitro and in vivo

**Fig. 2:**
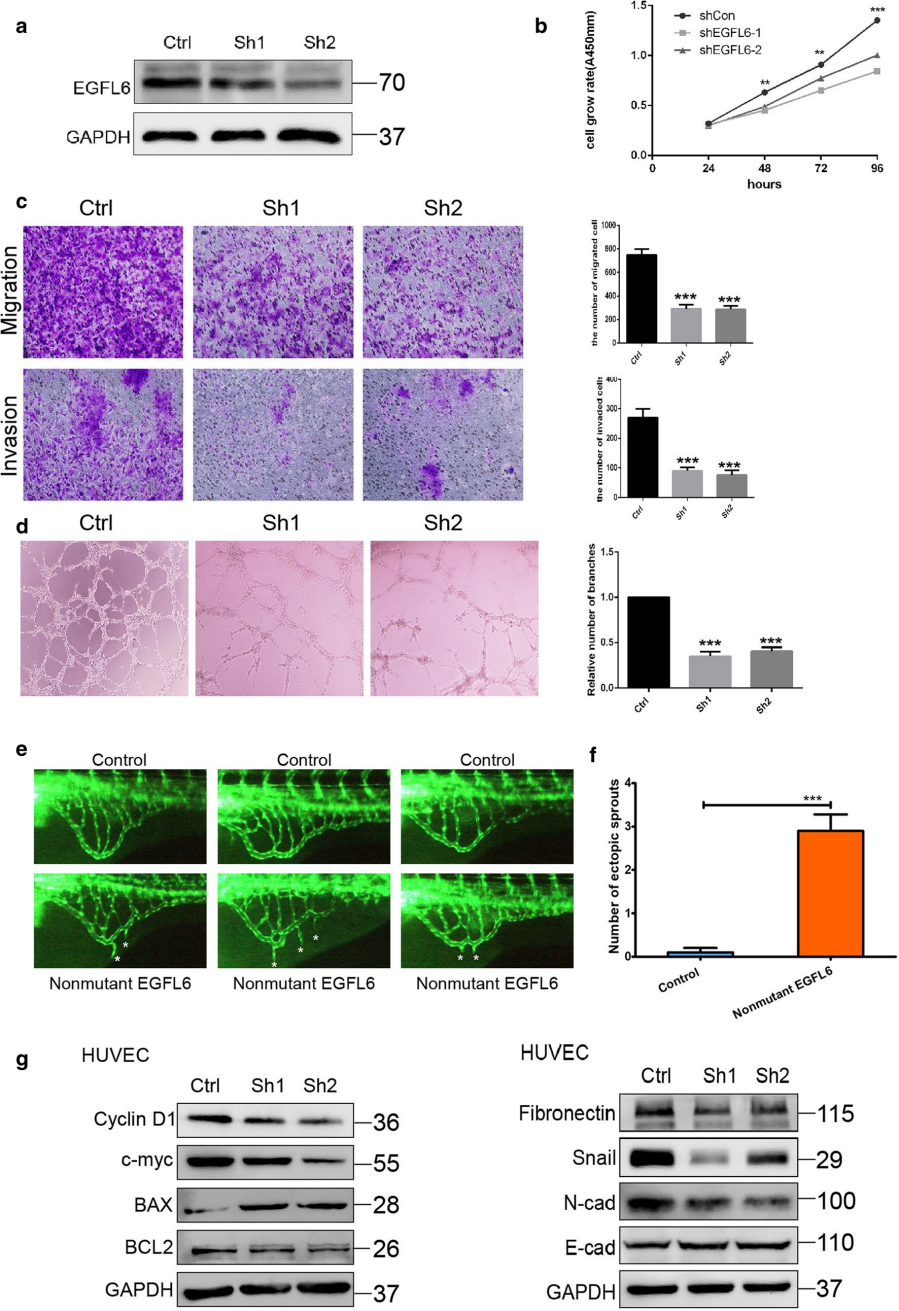
EGFL6 knockdown inhibits proliferation and angiogenesis in vivo and in vitro. **a** Western blot assay was used to determine the effect of EGFL6 siRNA. **b** cell viability was measured by CCK8 assay with HUVEC transfected with siNC and EGFL6 siRNA, Data are shown as the mean ± SEM. **c** cells migration and invasion assay with HUVEC transfected with siNC and EGFL6 siRNA, represented image was shown on the left and mean ± SEM was shown by bar chart. **d** Tuber formation assay with HUVEC transfected with siNC and EGFL6 siRNA, represented image was shown on the left and Mean ± SEM was shown by bar chart, *P < 0.05, **P < 0.01. **e** Representative fluorescent images of uninjected control or embryos injected with 100 pg nonmutant human *EGFL6* mRNA at 3,dpf. The subintestinal vessels form on the dorsolateral surface of the yolk on both sides of the embryo in the shape of a basket over the yolk. Compared with control, higher magnification of the SIV revealed that ectopic branches (asterisk) can be observed after nonmutant human *EGFL6* mRNA injection, dpf, days postfertilization; SIV, subintestinal vein. **f** Quantification of the average number of ectopic SIV segments. ***P < 0.001 (*n* = 10; Student’s *t* test). **g** The expressions of molecules related with cell proliferation (left) and cell migration (right) were detected with western blot. Data represent the average of three independent experiments

To test our hypothesis that EGFL6 is related to angiogenesis, we knocked down EGFL6 in HUVECs using *EGFL6* siRNA. First, the effect of the knockdown was verified by western blotting (Fig. 2a). Next, we conducted a CCK8 assay to determine the change in HUVEC viability after knocking down EGFL6 expression. The results showed that the downregulation of EGFL6 can inhibit the proliferation of HUVECs (Fig. 2b). The results of Transwell^®^ invasion and migration assays suggested that HUVECs with low expression of EGFL6 showed weaker ability to penetrate the chamber compared with controls (Fig. 2c). In the tuber formation assay, knocking down EGFL6 expression suppressed the ability of HUVECs to undergo angiogenesis (Fig. 2d). To test the hypothesis in vivo, we generated an angiogenesis model in zebrafish overexpressing EGFL6 by injecting 100 pg of an EGFL6-expressing plasmid into 3-day-old zebrafish embryos. Figure 2e, f shows distinct sprouting angiogenesis of the subintestinal veins in zebrafish overexpressing EGFL6 compared with the control. In addition, we analyzed the expression of those proteins associated with cell proliferation and EMT, which showed that knocking down EGFL6 expression reduces the expression of proteins that regulate cell growth such as cyclin D1, c-myc, and BCL2 (Fig. 2g). At the same time, proteins such as Snail and E-cadherin, which are related with EMT, were downregulated after knocking down EGFL6 expression (Fig. 2g). Taken together, these results demonstrate that EGFL6 promotes angiogenesis of HUVECs by interfering with their proliferation, invasion, and migration.

### PAX6 promotes angiogenesis and regulates the function of EGFL6 in vitro and in vivo

**Fig. 3:**
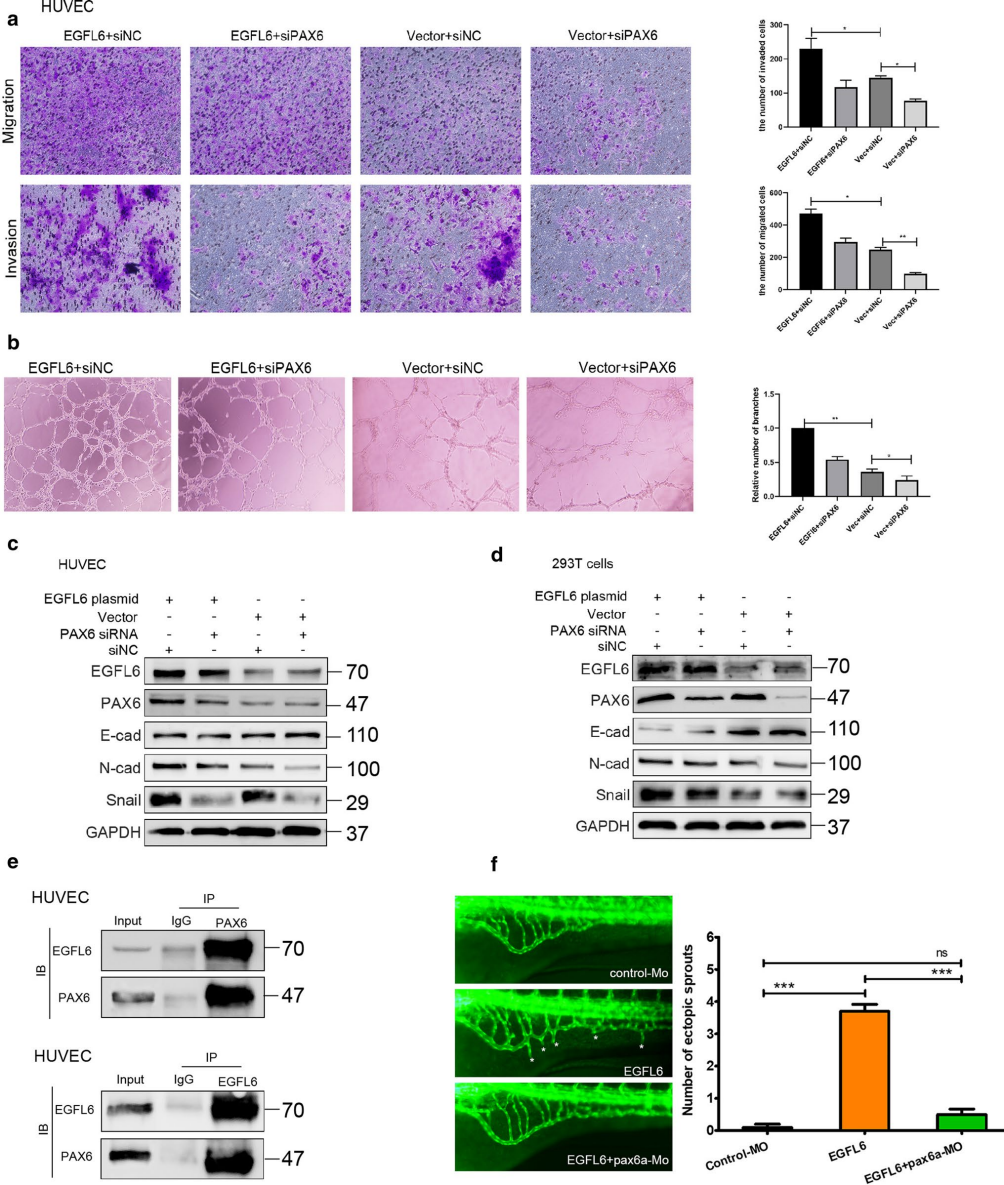
PAX6 is involved in EGFL6-mediated angiogenesis in vitro and in vivo. **a** cells’ migration and invasion assay with HUVEC that were treated with EGFL6 plasmid combined with PAX6 siRNA; represented image was shown on the left and Quantitative data are expressed as mean ± SEM, **P < 0.01, ***P < 0.001. **b** Tuber formation assay with HUVEC that were treated with EGFL6 plasmid combined with PAX6 siRNA; represented image was shown on the left and result was shown by bar chart, **P < 0.01, ***P < 0.001. **c**, **d** The expression of molecules related with cell migration in HUVEC and HEK293T cells that were treated with EGFL6 plasmid combined with PAX6 siRNA were detected with western blot. **e** Interaction between endogenous PAX6 and EGFL6 was detected by co-immunoprecipitation assay with anti-EGFL6 and anti-PAX6 antibody in HUVEC. The immunoglobulin G (IgG) antibody bought from BioSharp company was used as the control group and cell lysates were exploited to examine the expression of EGFL6 and PAX6. **f** Representative fluorescent images of zebrafish embryos at 72 hpf treated with control-MO (4 ng per embryo), EGFL6 (100 pg per embryo), or EGFL6 plus pax6a-MO (4 ng per embryo). Compared with EGFL6 overexpression embryos, embryos co-administered with pax6a-MO and EGFL6 present a decreased number of ectopic SIV segments (asterisk), quantification of the average number of ectopic SIV segments at 72 hpf. (*n* = 10; ANOVA; ***P < 0.0001; *ns* not significant.). Data represent the average of three independent experiments

To explore the mechanism of EGFL6 in angiogenesis, we divided HUVECs into EGFL6 knockdown and control groups and subjected these to RNA sequencing. The results showed that 25 genes were involved in the angiogenesis-promoting effect of EGFL6 (Supplementary Fig. 1a and 1b). Among these, we found that PAX6 was significantly affected by knocking down EGFL6. To test the correlation between EGFL6 and PAX6, a series of assays were performed. First, we constructed an HUVEC model with diminished EGFL6 levels using the EGFL6 siRNA, and measured the expression of *PAX6* mRNA and protein by RT-PCR and western blotting. The results suggested that the expression of PAX6 is mediated by EGFL6 (Supplementary Fig. 1c and 1d). In addition, the results of IHC and the immunofluorescence assay clearly indicated that the PAX6 protein is correlated and colocalizes with the EGFL6 protein in SBVM tissues (Supplementary Fig. 1e and 1f). To investigate the function of PAX6 in SBVM, we examined the expression of the PAX6 protein in SBVM tissues, and found that it was significantly higher compared to the expression in normal tissues (Supplementary Fig. 2a). Furthermore, we performed loss of function assays in HUVECs. The transfection efficiency of PAX6 siRNA was evaluated by RT-PCR and western blotting (Supplementary Fig. 2b). The Transwell^®^ assay indicated that the knockdown of *PAX6* in HUVECs significantly reduced both cell migration and invasion capacity (Supplementary Fig. 2c). In addition, the result of tuber formation assays strongly suggested that PAX6 is essential to angiogenesis (Supplementary Fig. 2d). Western blotting indicated that the knockdown of PAX6 reduced EMT markers such as Snail, fibronectin, and N-cadherin and increases E-cadherin expression (Supplementary Fig. 2e). To build an in vivo assay, we used a zebrafish pax6a morpholino, focusing on specific morpholino antisense strategies to prevent the proper splicing of exon 4 (E4I4,MO). We injected 4 ng of the specific morpholino antisense into zebrafish embryos. Embryos injected with pax6a-MO resulted in specific defects in the subintestinal vein vessels’ (SIV) formation (Supplementary Fig. 2f). Hence, these findings demonstrated that the PAX6 promoted angiogenesis of HUVECs by altering the capacity of invasion and migration of these cells. To better understand the association between EGFL6 and PAX6, we transfected *PAX6* siRNA into HUVEC combined with the EGFL6 plasmid and performed Transwell^®^ and tuber formation assays. The results showed that the knockdown of *PAX6* can reverse the effect of EGFL6 overexpression on the migratory capacity and angiogenic ability (Fig. 3a, b). At the same time, we conducted western blotting to test whether EMT markers would be altered, as predicted by our hypothesis. As expected, concordant to its functional consequences, the expression of EMT markers in HUVECs with EGFL6 overexpression was reversed following knockdown of *PAX6* (Fig. 3c, d). In a reciprocal Co-IP assay, we found that EGFL6 precipitated with PAX6, indicating that the endogenous human EGFL6 is physically related to PAX6 (Fig. 3e). In addition, we found that the angiogenesis-promoting effect of EGFL6 was impaired by knocking down PAX6 in a zebrafish model (Fig. 3f). Taken together, these data support the hypothesis that PAX6 promotes angiogenesis and regulates the function of EGFL6.

### Thalidomide inhibits angiogenesis by inducing degradation of EGFL6 in vitro and in vivo

**Fig. 4:**
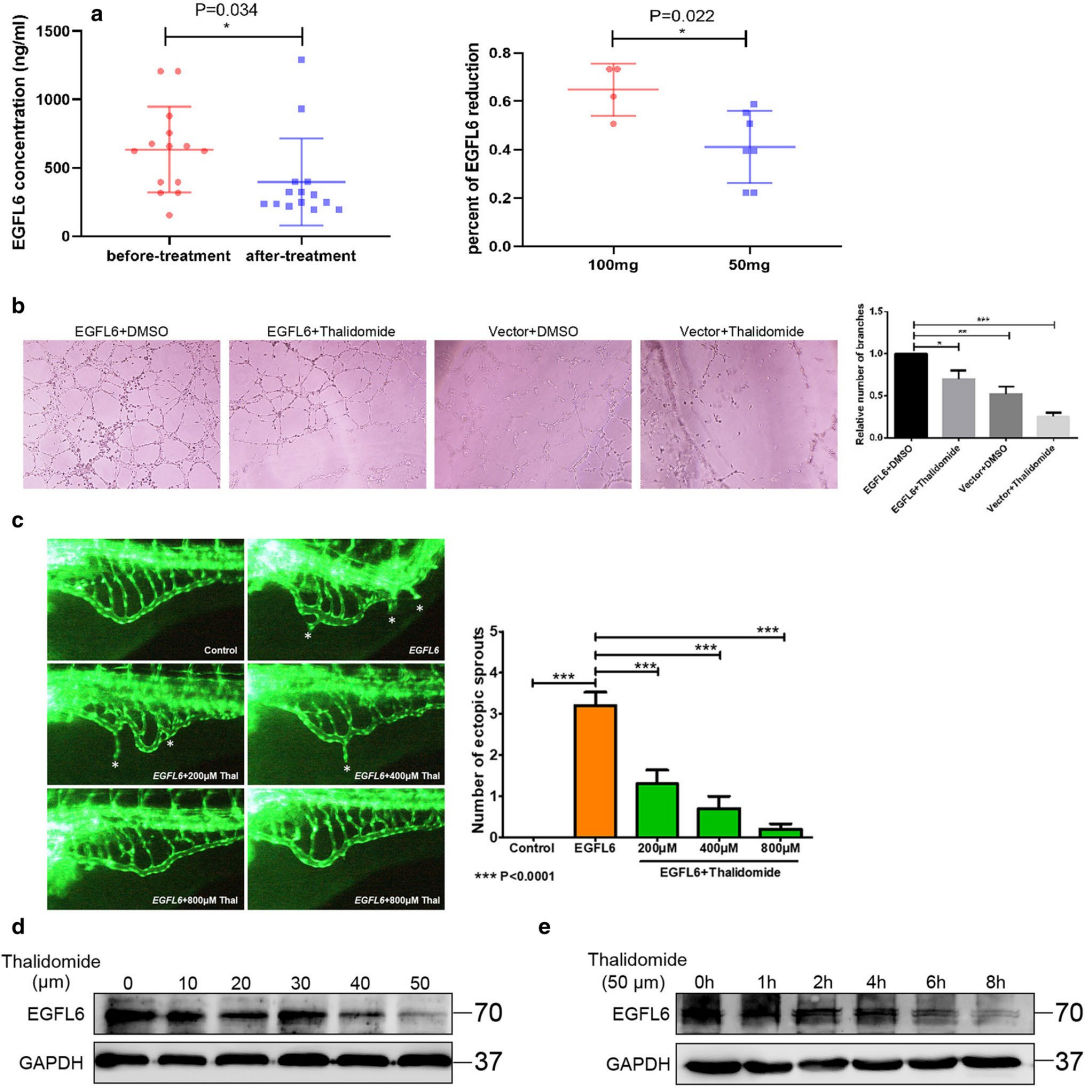
Thalidomide inhibits angiogenesis by targeting EGFL6 in vitro and in vivo. **a** ELISA assay was performed to detect the expression of EGFL6 in 14 SBVM patients who were treated with thalidomide; picture on the left was the alteration of EGFL6 expression between before treatment and after treatment; picture on the right was the percent of EGFL6 reduction between patients treated with 100 mg per day and patients treated with 50 mg per day. **b** Tuber formation assay with HUVEC that were treated with EGFL6 plasmid combined with 50 μM thalidomide; represented image was shown on the left; and result was shown by bar chart, **P < 0.01, ***P < 0.001. **c** Representative fluorescent images of zebrafish embryos at 72 hpf treated with 0.1% dimethyl sulfoxide (DMSO) (control) and *EGFL6* (200 pg per embryo) or co-administered with thalidomide (200 μM, 400 μM, 800 μM) for 24 h. Compared with *EGFL6* overexpression embryos, embryos’ treatment with thalidomide present a decreased number of ectopic SIV segments (asterisk), quantification of the average number of ectopic SIV segments at 72 hpf. *n* = 10; ANOVA; ***P < 0.0001. **d** HUVEC were treated with thalidomide at different concentration and western blot assay was performed to determine the expression of EGFL6. **e** HUVEC were treated with 50 μm thalidomide at different time point and cell lysate were subjected to western blot assay to determine the expression of EGFL6

**Fig. 5:**
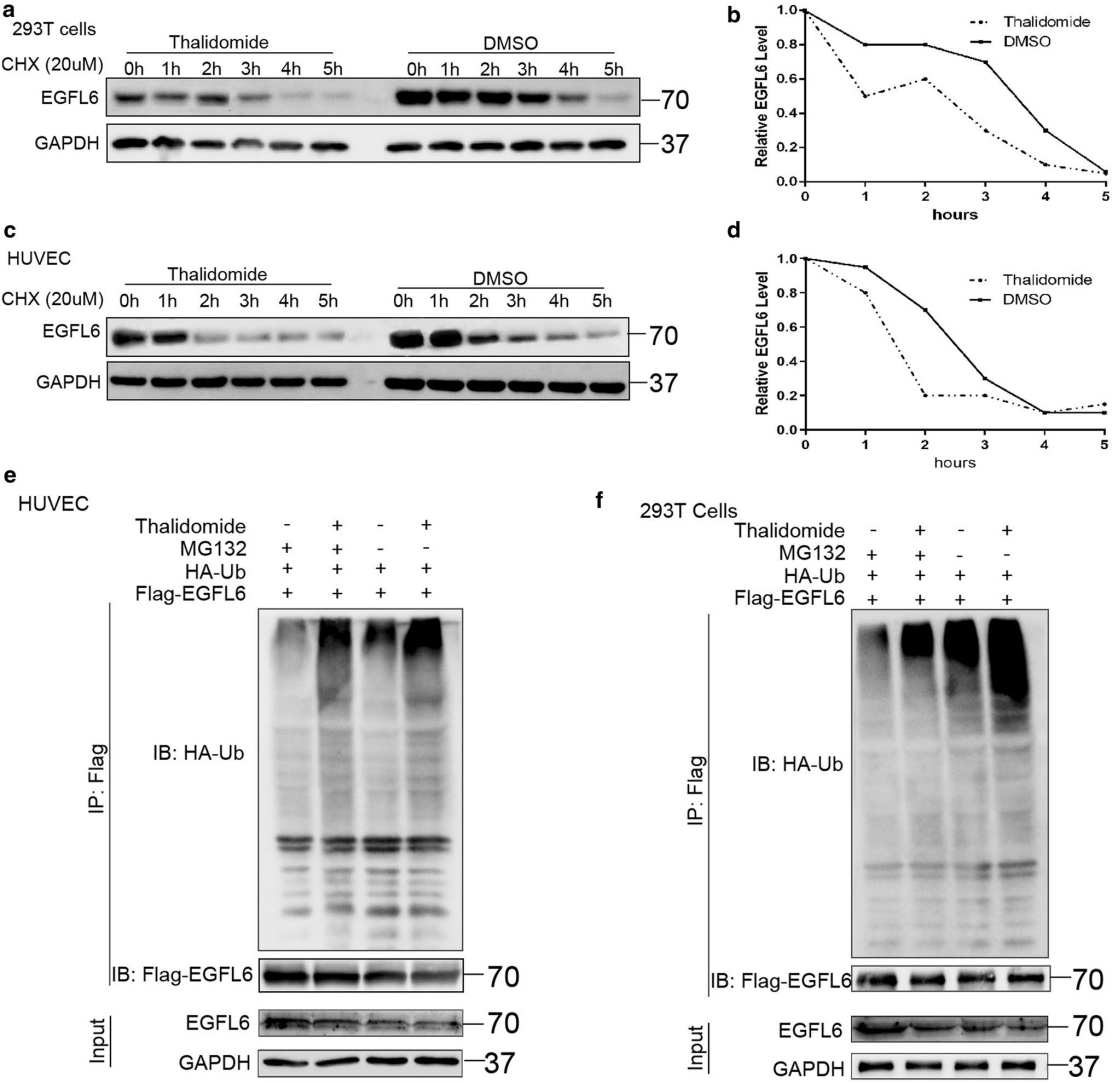
Thalidomide degrades EGFL6 by enhancing its ubiquitination level. **a**, **d** HEK293T cells and HUVEC were treated with 50 μm thalidomide combined with 20 μM CHX at indicated time. **a** HEK293T cells lysate were subjected to western blot assay to determine the expression of EGFL6. **b** The immunoblotting of EGFL6 was quantified by Image J. **c** HUVEC lysate were subjected to western blot assay to determine the expression of EGFL6. **d** The immunoblotting of EGFL6 was quantified by Image J. **e**, **f** HA-Ub and Flag-EGFL6 plasmids were co-transfected in HEK293T cells. After 48 h, cells were treated with 20 μm MG132 and 50 μm for 6 h. EGFL6 ubiquitination level was assessed by immunoprecipitation and EGFL6 expression after treatment of thalidomide and MG132 was detected by western blot

Our previous results demonstrated that EGFL6 is upregulated in SBVM and promotes angiogenesis in vivo and in vitro. Our team first demonstrated that thalidomide is an effective therapy for SBVM [5], but the specific targets of thalidomide have not been identified. To test our hypotheses, we collected samples from the 14 SBVM patients who had been treated with thalidomide for 4 months and followed up for a year (Table 1). We performed ELISAs and found the level of EGFL6 in the serum of patients before treatment was markedly higher before treatment, which suggested that thalidomide can reduce the level of EGFL6 expression (*P* = 0.034) (Fig. 4a). Furthermore, we divided these patients into two groups according to the given dose of thalidomide and found that the percentage of EGFL6 reduction was higher in patients treated with 100 mg thalidomide per day compared with those who were treated with 50 mg thalidomide per day (*P* = 0.022) (Fig. 4a). Combining these data with the analysis of bleeding events after thalidomide administration (Table 1), we hypothesize that thalidomide can treat SBVM by inhibiting angiogenesis through the modulation of the levels of EGFL6. In the tuber formation assay, when the HUVECs transfected with the EGFL6 plasmid were treated with 50 μM thalidomide, we found an inhibitory effect on tuber formation compared to the cells that were not given the drug (Fig. 4b). In addition, in the zebrafish model with EGFL6 overexpression, we found that thalidomide inhibited the abnormal proliferation of blood vessels due to overexpression of EGFL6, which was dependent on the dose of thalidomide (Fig. 4c). Notably, we found that the abnormal vessels were almost completely inhibited at 800 μM (Fig. 4c). We further tested the HUVECs in two ways: one was to treat the cells with different concentrations of thalidomide, and the other was to treat the cells with a fixed concentration of the drug (50 μM) for different periods. Western blotting was performed and we found that the EGFL6 protein expression decreased with thalidomide in both dose-dependent and time-dependent manners (Fig. 4d, e). To support our hypothesis that thalidomide degrades the EGFL6 protein, we treated HUVECs and HEK293T cells with thalidomide or DMSO, and a cycloheximide (CHX) pulse-chase assay was performed to examine the stability of the EGFL6 protein. Figure 5a, b shows that thalidomide treatment significantly increased the degradation of EGFL6 protein in HUVECs after using CHX to block protein synthesis. The same result was observed in HEK293T cells (Fig. 5c, d). Furthermore, an in vivo ubiquitination assay was performed, which showed that the ubiquitination level of EGFL6 increased with thalidomide treatment (Fig. 5e, f). We also found that treatment with proteasome inhibitors (MG132) alleviated the thalidomide-induced degradation of the EGFL6 protein (Fig. 5e, f). These findings suggest that thalidomide regulates EGFL6 in a proteasome-dependent manner.

**Table 1:** Clinical information of 14 patients who were treated with thalidomide

Patients	Height (cm)	Sex	Weight	Age	Total number of bleeding before treatment	Total number of bleeding during treatment	Total number of bleeding after treatment	Level of Hb before treatment (g/L)	Level of Hb after treatment	Level of EGFL6 before treatment	Level of EGFL6 after treatment	Dosage of thalidomide (mg/d)
Patient 1	169	M	62	43	4	0	0	107	143	1205.75	322	100
Patient 2	170	M	55	72	4	1	1	82	91	623.5	237	50
Patient 3	170	M	73	51	4	0	0	127	158	394.5	194.5	100
Patient 4	165	F	70	70	4	0	0	83	96	317	247	50
Patient 5	172	M	58	67	4	1	0	60	66	153.25	218.25	100
Patient 6	150	F	42	78	4	0	1	109	134	317	247	50
Patient 7	172	M	58	60	5	0	2	79	130	658.25	398.25	50
Patient 8	173	M	74	72	5	1	1	78	115	1205.75	322	100
Patient 9	142	F	49	45	4	0	0	89	138	754.5	930.75	50
Patient 10	158	F	53	43	4	0	0	110	123	658.25	398.25	50
Patient 11	172	M	65	56	4	0	1	79	157	623.25	237	100
Patient 12	164	F	63	70	4	2	0	93	107	394.5	194.5	50
Patient 13	158	F	49	58	5	0	0	75	136	675.75	302	50
Patient 14	169	F	65	66	4	1	0	93	110	879.5	1289.5	100

### E3 ubiquitin ligase cereblon efficiently regulates thalidomide-induced degradation of EGFL6

**Fig. 6:**
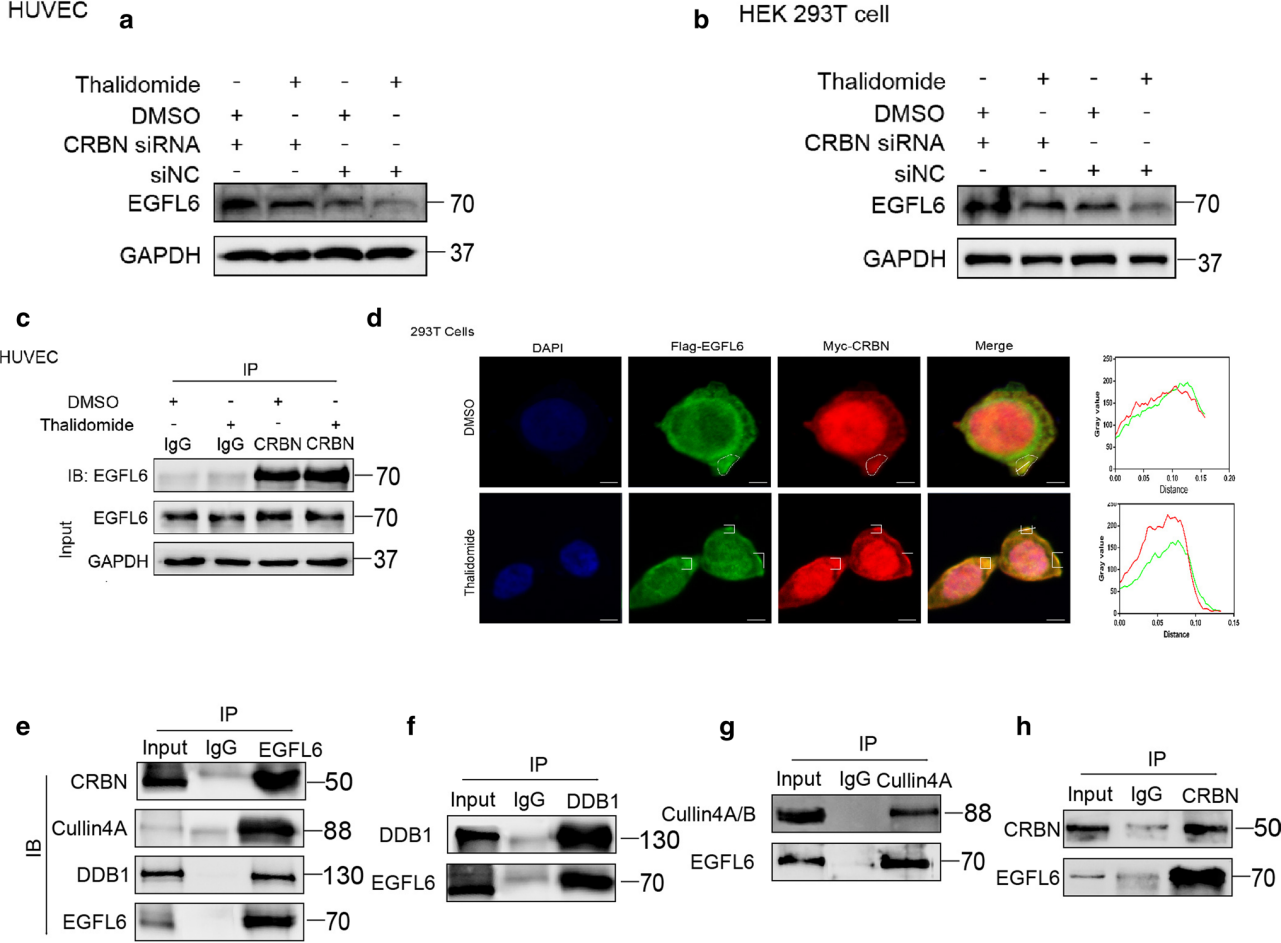
CRBN regulates the degradation of EGFL6 induced by thalidomide. **a**, **b** HEK293T cells and HUVEC were treated with CRBN siRNA combined with 50 μM thalidomide; western blot was performed to determine the expression of EGFL6. **c** HUVEC were treated with 50 μM thalidomide. Co-immunoprecipitataion assay was performed to explore the interaction between EGFL6 and CRBN. **d** HUVEC were transfected with Flag-EGFL6 plasmid and Myc-CRBN plasmid, after 48 h, cells were treated with thalidomide. Immunofluorescence assay was performed to detect the localization between EGFL6 and CRBN. **e**, **h** Co-immunoprecipitataion assay was performed to determine the interaction between EGFL6 and CRBN–DDB1–Cullin 4A complex

**Fig. 7:**
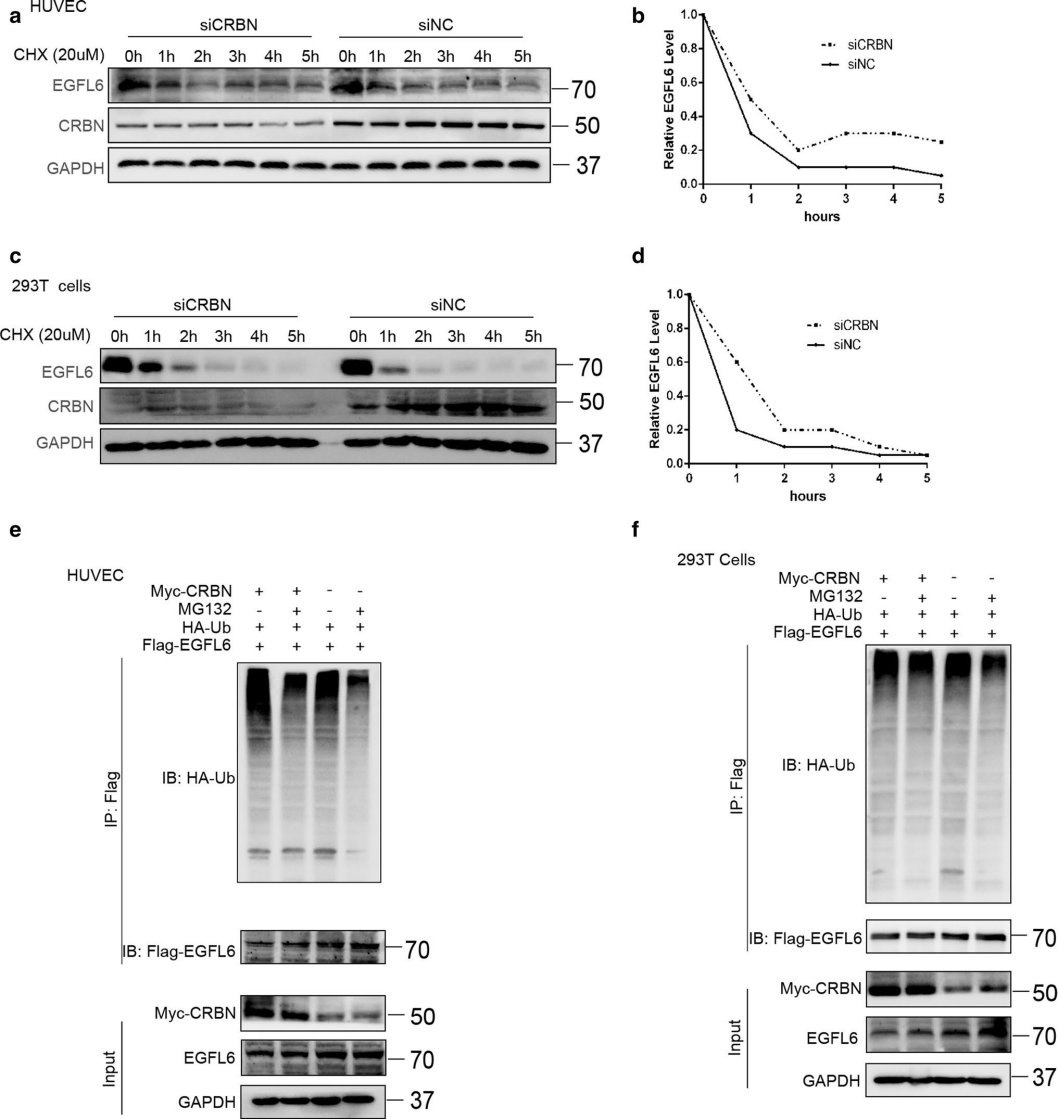
CRBN degrades EGFL6 by enhancing its ubiquitination level. **a**, **d** HEK293T cells and HUVEC were treated with CRBN siRNA combined with 20 μM CHX at indicated time. **a** HUVEC lysate were subjected to western blot assay to determine the expression of EGFL6. **b** The immunoblotting of EGFL6 was quantified by Image J. **c** HEK293 cells lysate were subjected to western blot assay to determine the expression of EGFL6. **d** The immunoblotting of EGFL6 was quantified by Image J. **e**, **f** HA-Ub plasmid, Flag-EGFL6 plasmid and CRBN siRNA were co-transfected in HEK293T cells. After 48 h, cells were treated with 20 μm MG132 for 6 h. EGFL6 ubiquitination level was assessed by immunoprecipitation and EGFL6 expression was detected by western blot

**Fig. 8:**
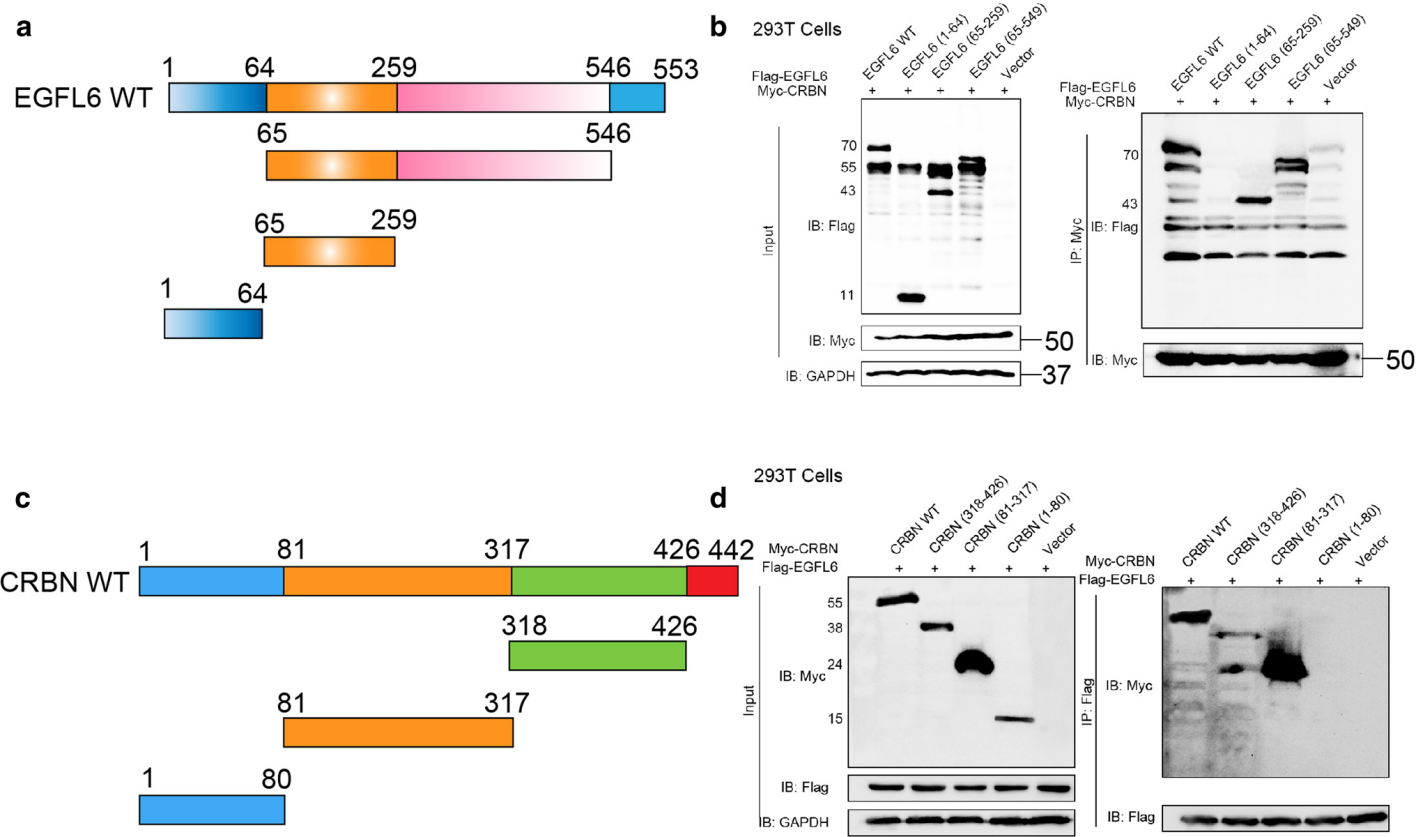
CRBN interacts with EGFL6. **a** A schematic diagram shows the structural domains of EGFL6 according to Uniprot website. **b** The control vector, EGFL6 wide-type (WT) plasmid, and truncated mutants of EGFL6 were co-transfected with Myc,CRBN WT plasmid into HEK293T cells. Co-immunoprecipitataion assay was performed to detect the interaction between EGFL6 and CRBN protein. **c** A schematic diagram shows the structural domains of CRBN according to Uniprot website. **d** The control vector, CRBN WT plasmid, and truncated mutants of CRBN were co-transfected with Flag,EGFL6 WT plasmid into HEK293T cells. Co-immunoprecipitataion assay was performed to detect the interaction between EGFL6 and CRBN proteins

Previously, it was reported that thalidomide can affect the binding of substrate CUL4–DDB1–CRBN ubiquitin ligase by directly binding to CRBN, which in turn regulates the ubiquitination of substrate proteins [17–19]. To investigate whether CRBN mediates the function of thalidomide, we transfected CRBN siRNA to knock down CRBN expression in combination with treatment of thalidomide. The results showed that the downregulation of CRBN notably enhanced the expression of EGFL6 and impaired the effect of thalidomide on EGFL6 expression (Fig. 6a, b). We then investigated whether thalidomide can promote the binding between EGFL6 and CRBN. As the results of CoIP and immunofluorescence assay showed, the interaction between EGFL6 and CRBN was enhanced in the presence of thalidomide (Fig. 6c, d). Moreover, endogenous EGFL6 could interact with CUL4-DDB1-CRBN ubiquitin ligase, as verified by CoIP assay in HEK293T cells (Fig. 6e, h). We depleted the expression of CRBN by CRBN siRNA and combined it with a CHX treatment to block protein production in HEK293T cells and HUVECs. We then measured the alteration of EGFL6 degradation and found that the rate of EGFL6 degradation significantly decreased when CRBN was knocked down (Fig. 7a, d). Additionally, the EGFL6 protein level also obviously increased with the addition of the MG132 proteasome inhibitor in HEK293T cells and HUVECs (Fig. 7e, f). Overexpression of CRBN increased the ubiquitination of EGFL6, allowing EGFL6 to proceed into the degradation process, which was inhibited by MG132 (Fig. 7e, f). To investigate the interaction of the CRBN and EGFL6 proteins, we constructed plasmids expressing truncated versions of EGFL6 and CRBN based on the domains of the proteins described in the UniProt website (Fig. 8a, c). A reciprocal CoIP assay was then performed to define the interaction. We found that only wild-type EGFL6 and the truncated plasmid encoding 194 amino acids (65–259) maintained the interaction with CRBN, and only wild-type CRBN and the truncated plasmid encoding 236 amino acids (81-317, Lon N-terminal) could keep the interaction with EGFL6 (Fig. 8b, d).

## Discussion

SBVM, which is characterized by ecstatic blood vessels made of thin walls with or without endothelial lining, is the major cause of OGIB in patients above 40 years of age [20]. Clinically, it is difficult to establish a diagnosis in patients with obscure bleeding, and thus, these individuals eventually require blood transfusions and repeated hospitalization [20]. Therefore, the medical cost for managing OGIB is fairly high. Moreover, for several years, our team has been investigating the pathogenesis of this disease, and we have found that thalidomide can cure the obscure bleeding caused by SBVM [5]. Further improvement in the treatment and management of patients with SBVM requires an in-depth understanding of the pathogenesis of SBVM and the mechanism of thalidomide. Here, we have shown that thalidomide targets EGFL6 to undergo proteasome degradation and inhibits the angiogenesis induced by EGFL6 overexpression in SBVM. The CRBN protein is involved in thalidomide-induced degradation of EGFL6. Overexpression of EGFL6 induced the development of abnormal subintestinal vein vessels in a zebrafish model, a process that was impaired by knocking down *PAX6* or treatment with thalidomide. These findings established that thalidomide regulates EGFL6 expression through proteasome degradation to inhibit the EGFL6/PAX6 axis-driven angiogenesis in SBVM.

Based on our previous studies on the pathogenesis of SBVM and the implication of other studies on the function of EGFL6 in angiogenesis [3, 12, 14], we performed some assays to investigate the specific mechanism of EGFL6 in SBVM. First, using the SBVM samples and by collecting the serum from patients with SBVM, we found that EGFL6 was upregulated compared to that in the serum of healthy controls. Compared to EGFL6 levels in tumors, it was found EGFL6 was overexpressed in lung cancer, colorectal cancer, breast cancer, nasopharyngeal cancer, ovarian cancer, and hepatocellular carcinoma [10, 11, 21–23]. Among these, it has been found EGFL6 promotes the angiogenesis in the development of tumor [10, 11]. To some extent, EGFL6 may play the same role in the process of angiogenesis no matter for tumorigenesis or SBVM, suggesting that EGFL6 could be overexpressed in SBVM and tumor. As for the level of EGFL6 in serum, two studies have reported EGFL6 level was highly expressed in nasopharyngeal carcinoma (120 pg/ml vs. 46 pg/ml or so) [9] and oral squamous cell carcinoma (304.48 ± 194.55 pg/mL vs. 178.69 ± 102.96 pg/mL; *p* < 0.001) [24]. In addition, a study analyzed the serum EGFL6 levels in healthy controls and patients with various tumors. It found the levels in healthy controls ranged from 0 to 48 pg/ml, while the levels in patients with various tumors ranged from 0 to 1364 pg/ml, of which the level of EGFL6 in ovarian cancer was highest (616 pg/ml) [25]. While in our study, we found the level of EGFL6 in SBVM was 632.9 pg/ml, which was higher than that in healthy controls. The reasons leading to the inconsistent results may be due to the different ELISA test kits. In conclusion, EGFL6 levels are lower in the healthy controls and higher in the tumors or SBVM, which may help us to diagnose and monitor the progress of the disease.

Using a zebrafish model, we found EGFL6 facilitated the growth of subintestinal veins. However, the mechanism by which EGFL6 promotes the formation of new blood remains unclear. To address this question, we performed RNA-Seq to detect the downstream factors of EGFL6 and found PAX6 is a potential target protein. In fact, the previous reports have revealed that PAX6 is correlated with angiogenesis, wherein it regulates the differentiation of endothelial cells and is associated with the expression of VEGF [26–28]. Our in vitro and in vivo assays suggest that PAX6 is essential for the process of angiogenesis of HUVECs by regulating the process of EMT, which agrees with the previously described consequence of overexpressing PAX6 for the pathogenesis of human cancers [29, 30]. In an attempt to rescue the EGFL6-driven abnormal angiogenesis, we found that PAX6 depletion impaired the vascular malformation (Fig. 6f). To date, although there has been no direct evidence to demonstrate that EGFL6 is correlated with PAX6, a study has shown that PAX6 regulates ITGB3 expression as a transcription factor [30]. Moreover, the members of the integrin family are the downstream targets of signaling cascades induced by EGFL7. EGFL7 promotes angiogenesis by enhancing the surface expression of integrins such as integrin α5β1 and integrin αVβ3; however, why the expression of the integrin family is upregulated is unclear [31–33]. Thus, the previous studies have suggested that members of the EGFL family may be correlated to PAX6 expression. Together with results of our current study, we may conclude that EGFL6 is an indispensable regulator of angiogenesis of HUVECs by targeting the PAX6 protein.

Thalidomide treatment is an emerging paradigm in SBVM [5]. Nevertheless, whether some proteins contribute to the therapeutic effect in SBVM remains elusive. Here, we constructed a zebrafish model overexpressing EGFL6 and we observed that vascular regeneration induced by EGFL6 overexpression was blocked in zebrafish that was treated with thalidomide but not in the control group (Fig. 7c). Furthermore, in the serum of patients treated with thalidomide, we found that the level of EGFL6 in the serum significantly decreased, which in coincides with the results of assays performed in vitro. Similarly, some studies have shown that thalidomide can downregulate the expression of secreted factors such as bFGF, FGF10, and VEGF [5, 34, 35]. Immunofluorescence co-localization and Co-IP experiments using truncated forms of EGFL6 and CRBN suggest that thalidomide can enhance the interaction between EGFL6 protein and CRBN. Furthermore, the Lon N-terminal peptide of CRBN was shown to be the binding site mediating the interaction with EGFL6. As described in other studies, CRBN binds to some proteins such as IKZF1/IKZF3 and MEIS2 via its Lon N-terminal peptide, which results in the degradation of the target proteins by the proteasome pathway [19, 36].

## Conclusions

Collectively, our findings provide novel insights into the mechanism underlying vessel malformation in SBVM induced by the increased expression of EGFL6 that promotes the EMT pathway via EGFL6/PAX6 signaling in HUVECs. Furthermore, we show that the therapeutic effect of thalidomide is due to the promotion of EGFL6 degradation by ubiquitination, which results from the thalidomide-dependent enhancement of EGFL6–CRBN protein interactions.

## Electronic supplementary material

Below is the link to the electronic supplementary material.

Supplementary material 1 (DOCX 29 kb)

Supplementary material 2 (DOCX 20 kb)

Supplementary material 3 (DOCX 899 kb)

Supplementary material 4 (DOCX 288 kb)

## Abbreviations

SBVM: Small bowel vascular malformation disease
HIF: Hypoxia induce factor
EGFL6: EGF like domain multiple 6
EMT: Epithelial–mesenchymal transition
UPS: Ubiquitination protease system
CRBN: Cereblon
PAX6: Paired box 6
VEGF: Vascular endothelial growth factor
IKZF3: IKAROS family zinc finger 3
FGF10: Fibroblast growth factor 10
