# Supplementary material for: Thalidomide targets EGFL6 to inhibit EGFL6/PAX6 axis-driven angiogenesis in small bowel vascular malformation
Source: Cell Mol Life Sci. 2020 Feb 1;77(24):5207–21. doi: 10.1007/s00018-020-03465-3 (PMC7671996; doi:10.1007/s00018-020-03465-3)
Supplement: Supplementary file 1 — Supplementary material 1 (DOCX 29 kb) [file 18_2020_3465_MOESM1_ESM.docx]

**Patients and Samples**

We used eight paraffin-embedded SBVM samples collected from patients in the Renji hospital who underwent surgery due to massive bleeding. Available serum samples from 14 patients treated with thalidomide (Changzhou Pharmaceutical Company, China) were used to analyze the expression of EGFL6 and its correlation with hemoglobin (Hb). The course of treatment was 4 months and the follow-up time was 12 months. All patients were provided informed consent by the Biomedical Ethics Committee of Renji Hospital. The detailed information about patients is shown in Table 1.

**Zebrafish**

As shown in our previous study,^1^ during care and maintenance adult zebrafish were maintained at 28.5°C on a 14 h light/10 h dark cycle. Five to six pairs of zebrafish were set up for natural mating every time. On average, 200–300 embryos were generated. Embryos were maintained at 28.5°C in fish water (0.2% Instant Ocean Salt in deionized water). Our experiment was allowed by The Shanghai Research Center For Model Organisms Animal Care and Use Committee. For the over-expression assay, fertilized one-cell embryos were injected with 100 pg of pcDNA3 encoding the human *EGFL6* cDNA (nonmutant) at a concentration of 100 ng/μL. The coding region of the wild-type human *EGFL6* cDNA was synthesized by Sangon Biotech and subcloned into pcDNA3.1 vector (Invitrogen). For the *PAX6a* gene knock-down experiment, 4 ng of control-MO or pax6a-e4i4-MO (E4I4-MO) were used per injection. Thalidomide (Sigma-Aldrich, St Louis, MO) was dissolved in dimethylsulfoxide (DMSO) at a stock concentration of 800 mM. Control embryos were treated with the equivalent amount of DMSO solution. Image acquisition and statistical analysis were performed as in a previous study.^1^

**Cell culture**

HUVECs were purchased from ATCC and HEK293T was obtained from the American Type Culture Collection (Manassas, VA, USA) (supplementary material 2). According to the introduction, HUVECs were cultured in the Endothelial Cell Medium (ECM) (ScienCell, USA) medium, and the HEK293T cells were cultured in Dulbecco’s Minimal Essential Medium (DMEM) (Gibco, Gaithersburg, MD, USA), supplemented with 10% fetal bovine serum (FBS; Gibco). Both cell lines were grown in an incubator at 37°C and in a 5% CO2 humidified environment. For hypoxia treatment, cells were incubated in a hypoxia chamber under 1% O_2_ after transfection for 24 hours or directly.

**Cell transfection**

EGF6 shRNAs, PAX6 shRNAs, and CRBN shRNAs were synthesized by GenePharm (Shanghai, China). In addition, we constructed an EGFL6^WT^ plasmid tagged with Flag and CRBN^WT^ tagged with Myc, and then, according to the information about EGFL6 and CRBN protein provided by the Uniprot website, we constructed the respective truncated plasmid by Asia-Vector Biotechnology Generay company (Shanghai, China). pcDNA3.1 was used as empty vector. The transfection reagent was FuGENE® HD Transfection Reagent (32043, Active Motif, USA). The detailed sequences of the proteins are shown in the supplementary material 3.

**Drug treatment**

For analyzing protein stability, HEK293T cells were treated with Cycloheximide (CHX) (40uM) (C7698, Sigma). For the ubiquitination assay, we treated the cells with the proteasome inhibitor MG132 (20 μM) (S2619) (Selleck inhibitors). Additionally, to investigate the effect of thalidomide, the drug was purchased from Sigma-Aldrich company (T144, USA). CHX, MG132, and thalidomide were dissolved in DMSO.

**Western blot analysis**

After transfection with siRNA or plasmid for around 48 h, cells were lysed by RIPA buffer containing three kinds of protein inhibitor (WeiAo, Shanghai). The total proteins were measured by the BCA Protein Assay Kit (Thermo Fisher Scientific, 23227, USA), and were subjected to 10% SDS-PAGE, and then transferred to Nitrocellulose Blotting Membrane (Pall Corporation, Port Washington, NY, USA). Finally, the membranes were blocked by 5% milk and incubated with the primary antibody at 4°C overnight. On the second day, membranes were washed five times and incubated with a secondary antibody (WeiAo, Shanghai), and the protein bands were detected using a ChemiDoc^TM^ Imaging System (Bio-Rad, Hercules, CA, USA). The main primary antibodies used in the study were: EGFL6 (1:1000, orb78333, Biorbyt), PAX6 (1:1000, ab5790), DDDDK tag (1:2500, ab1162), Myc tag (1:1000, ab9132), CRBN (1:1000, ab98992), N-cadherin (1:1000, ab18203), E-cadherin (1:1000, ab1416), Cullin4A/CUL4A (1:1000, ab92554), Snail (1:1000,3879, CST), HIF1A (1:1000,36169, CST), DDB1 (1:1000, ab109027), Fibronectin (1:1000, 15613-1-AP, Proteintech), Ubiquitin(1:1000, Active Motif, USA, 39741), GAPDH (1:3000; KangChen, Shanghai, China, KC-5G5), and HA tag (1:1000, ab9110, abcam).

**Transwell invasion and migration assays**

As described in a previous study,^2^ an 8-μm transwell chamber was used to perform invasion and migration assays. 1 × 10^5^ HUVECs were seeded into the upper chamber covering in 40 μl of diluted Matrigel (BD Biosciences, USA), whereas 600 μl of medium with a 20% FBS was added into the lower chamber. After 48 h, we fixed the cells on the lower surface with 4% formaldehyde (WeiAo, Shanghai, China) and wiped the cells on the upper surface with a cotton swab. Then we stained cells with 0.1% crystal violet and washed with PBS. Finally, we counted the number of cells in different fields by microscopy.

**RNA sequence and data analysis**

Total RNA was isolated from each thymic sample using the standard TRIzol protocol (Invitrogen, Carlsbad, CA, USA). The RNA quality was examined by gel electrophoresis and with a Nanodrop spectrophotometer (Thermo, Waltham, MA, USA). For RNA sequencing, RNA samples from 6 biological replicates were separated into two independent pools, each comprised of three distinct samples at equal amounts. Strand-specific libraries were constructed using the TruSeq RNA sample preparation kit (Illumina, San Diego, CA, USA), and sequencing was carried out using the Illumina HiSeq X Ten instrument by the commercial service of Genergy Biotechnology Co. Ltd. (Shanghai, China). The raw data was delivered to NCBI’s Gene Expression Omnibius, and was handled by Perl, and data quality was checked by FastQC v0.11.2. Clean reads were aligned to the chicken genome (release: Gallus gallus 4.0) from NCBI using Bowtie, with one mismatch allowed. As described in a previous study,^3^ the expression of the transcripts was calculated by FPKM using Perl. Differentially expression transcripts (DETs) were determined using the MA-plot-based method with Random Sampling (MARS) model in the DEGseq package between different time points. Then DETs were chosen for function and signaling pathway enrichment analysis using GO and the KEGG database. RNA-seq data were deposited in NCBI's Gene Expression Omnibus (GEO) and are accessible through GEO Series accession number GSE133000. The significantly enriched pathways were determined when P < 0.05 and at least two affiliated genes were included.

**Quantitative reverse-transcription PCR (qRT-PCR)**

As in our previous study,^4^ total RNA was extracted by Trizol reagent (Takara, Tokyo, Japan). cDNA was synthesized according to the instructions of the manufacturers from Takara, and SYBR Green was used for real-time PCR. Each assay was measured in triplicate. The value of 2−ΔΔCt was used to estimate the fold differences. The sequences of EGFL and PAX6 primers were as follows: EGFL6 sense, sense (5′‐CTCCTACCTGACCTGCAACC‐3′) and antisense (5’‐GCCAGGGCATTGTTACTGTT‐3’); PAX6: Sense (5’-TGGGCAGGTATTACGAGACTG-3’), anti-sense (5’-ACTCCCGCTTATACTGGGCTA-3’); GAPDH was used as control.

**CCK8 assay**

Two thousand cells were seeded into each well of a 96-well plate. According to the introduction of the Cell Counting Kit (Dojindo Molecular Technologies, Japan), cells were transfected with EGFL6 siRNA or control siRNA using the FuGENE HD Transfection Reagent in 96-wells plate and cell viability was measured at the indicated time points (0h, 24h, 48h, 72h). The optical density (OD) at 450 nm (indicating the formation of formazan) was measured using a VERSA Max microplate reader (MDS Analytical Technologies).

**Tuber formation assay**

As described in a previous study,^1^ Matrigel (BD Biosciences) was thawed overnight at 4°C and then 150 μl (diluted 1: 1 with medium) was added into each well of a 48-well plate, and the plate was placed for 1 h at 37°C to solidify. Cells (3.5 X 10^4^) were seeded into the wells gently and without creating bubbles, and were observed under microscopy after 4–6 h at 37°C. The junctions of tubes were counted to assess the effect on tuber formation, which mimics the capillaries.

**ELISA analysis**

An ELISA was performed as described in the EGFL6 ELISA Kit (MBS074197, MyBioSource). First, we centrifuged serum for approximately 20 minutes at 1000 g within 30 minutes after collection. We added 50 μl of standard solution to each standard well, added 50 μl of sample to sample well, and added Sample Diluent 50 μl to each control well. Then 100 μl of HRP-conjugate reagent was added to each well and the plate was incubated for 60 min at 37°C. After that, we washed the plate and added staining solution to each well. Finally, we added 50 μl of stop solution and read the optical density (OD) at 450 nm. The concentrations were calculated according to a linear standard curve made by the concentration of standards and the mean value of OD.

**Luciferase reporter assays**

As in our previous study,^4^ 1,500 cells were seeded into a plate for luciferase assay. Luc-PairTM Duo-Luciferase Assay Kit 2.0 was purchased from GeneCopoeia (China). An EGFL6 promoter plasmid (1 μg) or a truncated promoter plasmid were co-transfected into cells with 1 μg firefly plasmids; after 24 h, the luciferase activity was examined using FlUOstar Omega. The vector pGL3 was used in the control group.

**Immunoprecipitation**

As in our previous study,^4^ cells were seeded in a 10 cm dish with a density of 1 X 10^5^ and were transfected with shRNA or plasmid. Protein A-Agarose was used to bind protein and antibody. The primary antibodies were the same as those listed before. The following processes were complicated as western blot.

**Immunofluorescence Assay**

As in our previous study,^4^ cells were co-transfected with the EGFL6 plasmid and CRBN plasmid for 24 h, and then were seeded into chamber slides with a density of 2,000 cells for each chamber for 24 h. Next, cells were fixed with 4% paraformaldehyde for 15 min and permeabilized in a 0.1% Triton X-100 solution at room temperature for 1 h. Finally, cells were incubated with a primary antibody overnight at 4°C and incubated with secondary antibody for 1 h. The image was observed under a fluorescence microscope at wavelengths of 488 and 594 nm. Micrographs were captured by means of confocal software (ZEN system 2012 Black Edition, Zeiss). Colocalization analysis was performed using the “colocalization” module of the ImageJ program The primary antibodies include DDDDK tag (1:200, ab1162), Myc tag (1:100, ab9132), PAX6 (1:50, ab78545), EGFL6(1:100, ab140079), and corresponding secondary antibodies were obtained from Thermo Fisher Scientific.

**Immunohistochemistry**

Paraffin-embedded human small intestinal vascular malformation tissue sections from the Renji hospital were used to perform an immunohistochemistry assay. Briefly, the sections were deparaffinized and rehydrated, and, after antigen retrieval and blocking, incubated with the primary antibody. The primary antibody includes anti-EGFL6 (1:100, ab140079), PAX6 (1:100, ab5790), HIF1A (1:100, 36169, Cell Signaling Technology) and CD31 (1:100, ab28364). The section was incubated with peroxidase-labeled secondary antibody and stained by DAB reagent. Imaging of vessels was observed under microscopy.

**Statistical analysis**

All data are presented as mean ± SEM. Statistical analysis and graphical representation of the data were performed using GraphPad Prism 7.0 (GraphPad Software, San Diego, CA). Image J (SpartanCoders, 1.52o) was used to analyze the intensity of colocalization. Statistical significance was performed using a Student’s t,test or an analysis of variance (ANOVA) as appropriate. Statistical significance is indicated by *, where P < 0.05, and ***, where P < 0.001.

1. Feng N, Chen H, Fu S, et al. HIF-1alpha and HIF-2alpha induced angiogenesis in gastrointestinal vascular malformation and reversed by thalidomide. Sci Rep 2016;6:27280.

2. Lin XL, Yang L, Fu SW, et al. Overexpression of NOX4 predicts poor prognosis and promotes tumor progression in human colorectal cancer. Oncotarget 2017;8:33586-33600.

3. Trapnell C, Roberts A, Goff L, et al. Differential gene and transcript expression analysis of RNA-seq experiments with TopHat and Cufflinks. Nat Protoc 2012;7:562-78.

4. Tang CT, Liang Q, Yang L, et al. RAB31 Targeted by MiR-30c-2-3p Regulates the GLI1 Signaling Pathway, Affecting Gastric Cancer Cell Proliferation and Apoptosis. Front Oncol 2018;8:554.
