# Supplementary material for: Thalidomide targets EGFL6 to inhibit EGFL6/PAX6 axis-driven angiogenesis in small bowel vascular malformation
Source: Cell Mol Life Sci. 2020 Feb 1;77(24):5207–21. doi: 10.1007/s00018-020-03465-3 (PMC7671996; doi:10.1007/s00018-020-03465-3)
Supplement: Supplementary file 2 — Supplementary material 2 (DOCX 20 kb) [file 18_2020_3465_MOESM2_ESM.docx]

| Gene | siRNA sequence |
| --- | --- |
| EGFL6 siRNA-1 | Sense (5’-3’)  GGAAGCUACUACUGCAAAUTT |
|  | Anti-Sense (5’-3’)  AUUUGCAGUAGUAGCUUCCTT |
| EGFL6 siRNA-2 | Sense (5’-3’)  GCAGGGAUAUAAAGGCAAUTT |
|  | Anti-Sense (5’-3’)  AUUGCCUUUAUAUCCCUGCTT |
| PAX6 siRNA-1 | Sense (5’-3’)ACUUCAACAGGACUCAUUUTT |
|  | Anti-Sense (5’-3’)  AAAUGAGUCCUGUUGAAGUGG |
| PAX6 siRNA-2 | Sense  (5’-3’)AAGAAUACAGGUAUGGUUUTT |
|  | Anti-Sense (5’-3’)  AAACCAUAACCUGUAUUCUUGC |
| CRBN siRNA-1 | Sense (5’-3’)  CCAGCAAGCUAAAGUGCAATT |
|  | Anti-Sense (5’-3’)  UUGCACUUUAGCUUGCUGGTT |
| CRBN siRNA-2 | Sense (5’-3’)  GCGACUUCGCUGUGAAUUATT |
|  | Anti-Sense (5’-3’)  UAAUUCACAGCGAAGUCGCTT |

EGFL6 WT plasmid

ATGCCTCTGCCCTGGAGCCTTGCGCTCCCGCTGCTGCTCTCCTGGGTGGCAGGTGGTTTCGGGAACGCGGCCAGTGCAAGGCATCACGGGTTGTTAGCATCGGCACGTCAGCCTGGGGTCTGTCACTATGGAACTAAACTGGCCTGCTGCTACGGCTGGAGAAGAAACAGCAAGGGAGTCTGTGAAGCTACATGCGAACCTGGATGTAAGTTTGGTGAGTGCGTGGGACCAAACAAATGCAGATGCTTTCCAGGATACACCGGGAAAACCTGCAGTCAAGATGTGAATGAGTGTGGAATGAAACCCCGGCCATGCCAACACAGATGTGTGAATACACACGGAAGCTACAAGTGCTTTTGCCTCAGTGGCCACATGCTCATGCCAGATGCTACGTGTGTGAACTCTAGGACATGTGCCATGATAAACTGTCAGTACAGCTGTGAAGACACAGAAGAAGGGCCACAGTGCCTGTGTCCATCCTCAGGACTCCGCCTGGCCCCAAATGGAAGAGACTGTCTAGATATTGATGAATGTGCCTCTGGTAAAGTCATCTGTCCCTACAATCGAAGATGTGTGAACACATTTGGAAGCTACTACTGCAAATGTCACATTGGTTTCGAACTGCAATATATCAGTGGACGATATGACTGTATAGATATAAATGAATGTACTATGGATAGCCATACGTGCAGCCACCATGCCAATTGCTTCAATACCCAAGGGTCCTTCAAGTGTAAATGCAAGCAGGGATATAAAGGCAATGGACTTCGGTGTTCTGCTATCCCTGAAAATTCTGTGAAGGAAGTCCTCAGAGCACCTGGTACCATCAAAGACAGAATCAAGAAGTTGCTTGCTCACAAAAACAGCATGAAAAAGAAGGCAAAAATTAAAAATGTTACCCCAGAACCCACCAGGACTCCTACCCCTAAGGTGAACTTGCAGCCCTTCAACTATGAAGAGATAGTTTCCAGAGGCGGGAACTCTCATGGAGGTAAAAAAGGGAATGAAGAGAAAATGAAAGAGGGGCTTGAGGATGAGAAAAGAGAAGAGAAAGCCCTGAAGAATGACATAGAGGAGCGAAGCCTGCGAGGAGATGTGTTTTTCCCTAAGGTGAATGAAGCAGGTGAATTCGGCCTGATTCTGGTCCAAAGGAAAGCGCTAACTTCCAAACTGGAACATAAAGCAGATTTAAATATCTCGGTTGACTGCAGCTTCAATCATGGGATCTGTGACTGGAAACAGGATAGAGAAGATGATTTTGACTGGAATCCTGCTGATCGAGATAATGCTATTGGCTTCTATATGGCAGTTCCGGCCTTGGCAGGTCACAAGAAAGACATTGGCCGATTGAAACTTCTCCTACCTGACCTGCAACCCCAAAGCAACTTCTGTTTGCTCTTTGATTACCGGCTGGCCGGAGACAAAGTCGGGAAACTTCGAGTGTTTGTGAAAAACAGTAACAATGCCCTGGCATGGGAGAAGACCACGAGTGAGGATGAAAAGTGGAAGACAGGGAAAATTCAGTTGTATCAAGGAACTGATGCTACCAAAAGCATCATTTTTGAAGCAGAACGTGGCAAGGGCAAAACCGGCGAAATCGCAGTGGATGGCGTCTTGCTTGTTTCAGGCTTATGTCCAGATAGCCTTTTATCTGTGGATGACGATTACAAGGATGACGACGATAAG GATTACAAGGATGACGACGATAAGGATTACAAGGATGACGACGATAAGTGA

EGFL6（1-64 aa）plasmid

ATGCCTCTGCCCTGGAGCCTTGCGCTCCCGCTGCTGCTCTCCTGGGTGGCAGGTGGTTTCGGGAACGCGGCCAGTGCAAGGCATCACGGGTTGTTAGCATCGGCACGTCAGCCTGGGGTCTGTCACTATGGAACTAAACTGGCCTGCTGCTACGGCTGGAGAAGAAACAGCAAGGGAGTCTGTGAAGCTACAGATTACAAGGATGACGACGATAAGGATTACAAGGATGACGACGATAAGGATTACAAGGATGACGACGATAAG TGA

EGFL6（65-546 aa）plasmid

ATGTGCGAACCTGGATGTAAGTTTGGTGAGTGCGTGGGACCAAACAAATGCAGATGCTTTCCAGGATACACCGGGAAAACCTGCAGTCAAGATGTGAATGAGTGTGGAATGAAACCCCGGCCATGCCAACACAGATGTGTGAATACACACGGAAGCTACAAGTGCTTTTGCCTCAGTGGCCACATGCTCATGCCAGATGCTACGTGTGTGAACTCTAGGACATGTGCCATGATAAACTGTCAGTACAGCTGTGAAGACACAGAAGAAGGGCCACAGTGCCTGTGTCCATCCTCAGGACTCCGCCTGGCCCCAAATGGAAGAGACTGTCTAGATATTGATGAATGTGCCTCTGGTAAAGTCATCTGTCCCTACAATCGAAGATGTGTGAACACATTTGGAAGCTACTACTGCAAATGTCACATTGGTTTCGAACTGCAATATATCAGTGGACGATATGACTGTATAGATATAAATGAATGTACTATGGATAGCCATACGTGCAGCCACCATGCCAATTGCTTCAATACCCAAGGGTCCTTCAAGTGTAAATGCAAGCAGGGATATAAAGGCAATGGACTTCGGTGTTCTGCTATCCCTGAAAATTCTGTGAAGGAAGTCCTCAGAGCACCTGGTACCATCAAAGACAGAATCAAGAAGTTGCTTGCTCACAAAAACAGCATGAAAAAGAAGGCAAAAATTAAAAATGTTACCCCAGAACCCACCAGGACTCCTACCCCTAAGGTGAACTTGCAGCCCTTCAACTATGAAGAGATAGTTTCCAGAGGCGGGAACTCTCATGGAGGTAAAAAAGGGAATGAAGAGAAAATGAAAGAGGGGCTTGAGGATGAGAAAAGAGAAGAGAAAGCCCTGAAGAATGACATAGAGGAGCGAAGCCTGCGAGGAGATGTGTTTTTCCCTAAGGTGAATGAAGCAGGTGAATTCGGCCTGATTCTGGTCCAAAGGAAAGCGCTAACTTCCAAACTGGAACATAAAGCAGATTTAAATATCTCGGTTGACTGCAGCTTCAATCATGGGATCTGTGACTGGAAACAGGATAGAGAAGATGATTTTGACTGGAATCCTGCTGATCGAGATAATGCTATTGGCTTCTATATGGCAGTTCCGGCCTTGGCAGGTCACAAGAAAGACATTGGCCGATTGAAACTTCTCCTACCTGACCTGCAACCCCAAAGCAACTTCTGTTTGCTCTTTGATTACCGGCTGGCCGGAGACAAAGTCGGGAAACTTCGAGTGTTTGTGAAAAACAGTAACAATGCCCTGGCATGGGAGAAGACCACGAGTGAGGATGAAAAGTGGAAGACAGGGAAAATTCAGTTGTATCAAGGAACTGATGCTACCAAAAGCATCATTTTTGAAGCAGAACGTGGCAAGGGCAAAACCGGCGAAATCGCAGTGGATGGCGTCTTGCTTGTTTCAGGCTTATGTCCAGATAGCCTTTTATCTGTGGATGACGATTACAAGGATGACGACGATAAGGATTACAAGGATGACGACGATAAGGATTACAAGGATGACGACGATAAG TGA

EGFL6（△65-259 aa）plasmid

ATGCCTCTGCCCTGGAGCCTTGCGCTCCCGCTGCTGCTCTCCTGGGTGGCAGGTGGTTTCGGGAACGCGGCCAGTGCAAGGCATCACGGGTTGTTAGCATCGGCACGTCAGCCTGGGGTCTGTCACTATGGAACTAAACTGGCCTGCTGCTACGGCTGGAGAAGAAACAGCAAGGGAGTCTGTGAAGCTACAGCTATCCCTGAAAATTCTGTGAAGGAAGTCCTCAGAGCACCTGGTACCATCAAAGACAGAATCAAGAAGTTGCTTGCTCACAAAAACAGCATGAAAAAGAAGGCAAAAATTAAAAATGTTACCCCAGAACCCACCAGGACTCCTACCCCTAAGGTGAACTTGCAGCCCTTCAACTATGAAGAGATAGTTTCCAGAGGCGGGAACTCTCATGGAGGTAAAAAAGGGAATGAAGAGAAAATGAAAGAGGGGCTTGAGGATGAGAAAAGAGAAGAGAAAGCCCTGAAGAATGACATAGAGGAGCGAAGCCTGCGAGGAGATGTGTTTTTCCCTAAGGTGAATGAAGCAGGTGAATTCGGCCTGATTCTGGTCCAAAGGAAAGCGCTAACTTCCAAACTGGAACATAAAGCAGATTTAAATATCTCGGTTGACTGCAGCTTCAATCATGGGATCTGTGACTGGAAACAGGATAGAGAAGATGATTTTGACTGGAATCCTGCTGATCGAGATAATGCTATTGGCTTCTATATGGCAGTTCCGGCCTTGGCAGGTCACAAGAAAGACATTGGCCGATTGAAACTTCTCCTACCTGACCTGCAACCCCAAAGCAACTTCTGTTTGCTCTTTGATTACCGGCTGGCCGGAGACAAAGTCGGGAAACTTCGAGTGTTTGTGAAAAACAGTAACAATGCCCTGGCATGGGAGAAGACCACGAGTGAGGATGAAAAGTGGAAGACAGGGAAAATTCAGTTGTATCAAGGAACTGATGCTACCAAAAGCATCATTTTTGAAGCAGAACGTGGCAAGGGCAAAACCGGCGAAATCGCAGTGGATGGCGTCTTGCTTGTTTCAGGCTTATGTCCAGATAGCCTTTTATCTGTGGATGACGATTACAAGGATGACGACGATAAGGATTACAAGGATGACGACGATAAGGATTACAAGGATGACGACGATAAG TGA

Green markeris FLAG tag

CRBN WT plasmid

ATGGAGCAGAAACTCATCTCTGAAGAGGATCTGGCCGGCGAAGGAGATCAGCAGGACGCTGCGCACAACATGGGCAACCACCTGCCGCTCCTGCCTGCAGAGAGTGAGGAAGAAGATGAAATGGAAGTTGAAGACCAGGATAGTAAAGAAGCCAAAAAACCAAACATCATAAATTTTGACACCAGTCTGCCGACATCACATACATACCTAGGTGCTGATATGGAAGAATTTCATGGCAGGACTTTGCACGATGACGACAGCTGTCAGGTGATTCCAGTTCTTCCACAAGTGATGATGATCCTGATTCCCGGACAGACATTACCTCTTCAGCTTTTTCACCCTCAAGAAGTCAGTATGGTGCGGAATTTAATTCAGAAAGATAGAACCTTTGCTGTTCTTGCATACAGCAATGTACAGGAAAGGGAAGCACAGTTTGGAACAACAGCAGAGATATATGCCTATCGAGAAGAACAGGATTTTGGAATTGAGATAGTGAAAGTGAAAGCAATTGGAAGACAAAGGTTCAAAGTCCTTGAGCTAAGAACACAGTCAGATGGAATCCAGCAAGCTAAAGTGCAAATTCTTCCCGAATGTGTGTTGCCTTCAACCATGTCTGCAGTTCAATTAGAATCCCTCAATAAGTGCCAGATATTTCCTTCAAAACCTGTCTCAAGAGAAGACCAATGTTCATATAAATGGTGGCAGAAATACCAGAAGAGAAAGTTTCATTGTGCAAATCTAACTTCATGGCCTCGCTGGCTGTATTCCTTATATGATGCTGAGACCTTAATGGACAGAATCAAGAAACAGCTACGTGAATGGGATGAAAATCTAAAAGATGATTCTCTTCCTTCAAATCCAATAGATTTTTCTTACAGAGTAGCTGCTTGTCTTCCTATTGATGATGTATTGAGAATTCAGCTCCTTAAAATTGGCAGTGCTATCCAGCGACTTCGCTGTGAATTAGACATTATGAATAAATGTACTTCCCTTTGCTGTAAACAATGTCAAGAAACAGAAATAACAACCAAAAATGAAATATTCAGTTTATCCTTATGTGGGCCGATGGCAGCTTATGTGAATCCTCATGGATATGTGCATGAGACACTTACTGTGTATAAGGCTTGCAACTTGAATCTGATAGGCCGGCCTTCTACAGAACACAGCTGGTTTCCTGGGTATGCCTGGACTGTTGCCCAGTGTAAGATCTGTGCAAGCCATATTGGATGGAAGTTTACGGCCACCAAAAAAGACATGTCACCTCAAAAATTTTGGGGCTTAACGCGATCTGCTCTGTTGCCCACGATCCCAGACACTGAAGATGAAATAAGTCCAGACAAAGTAATACTTTGCTTGTAA

Blue marker is MYC tag

CRBN (1-80aa) plasmid

ATGGAGCAGAAACTCATCTCTGAAGAGGATCTGGCCGGCGAAGGAGATCAGCAGGACGCTGCGCACAACATGGGCAACCACCTGCCGCTCCTGCCTGCAGAGAGTGAGGAAGAAGATGAAATGGAAGTTGAAGACCAGGATAGTAAAGAAGCCAAAAAACCAAACATCATAAATTTTGACACCAGTCTGCCGACATCACATACATACCTAGGTGCTGATATGGAAGAATTTCATGGCAGGACTTTGCACGATGACGACAGCTGTCAGGTGATTCCATAA

CRBN (81-317aa) plasmid

ATGGCCGGCGAAGGAGATCAGCAGGACGCTGCGCACAACATGGGCAACCACCTGCCGCTCCTGCCTGCAGAGAGTGAGGAAGAAGATGAAATGGAAGTTGAAGACCAGGATAGTAAAGAAGCCAAAAAACCAAACATCATAAATTTTGACACCAGTCTGCCGACATCACATACATACCTAGGTGCTGATATGGAAGAATTTCATGGCAGGACTTTGCACGATGACGACAGCTGTCAGGTGTGTACTTCCCTTTGCTGTAAACAATGTCAAGAAACAGAAATAACAACCAAAAATGAAATATTCAGTTTATCCTTATGTGGGCCGATGGCAGCTTATGTGAATCCTCATGGATATGTGCATGAGACACTTACTGTGTATAAGGCTTGCAACTTGAATCTGATAGGCCGGCCTTCTACAGAACACAGCTGGTTTCCTGGGTATGCCTGGACTGTTGCCCAGTGTAAGATCTGTGCAAGCCATATTGGATGGAAGTTTACGGCCACCAAAAAAGACATGTCACCTCAAAAATTTTGGGGCTTAACGCGATCTGCTCTGTTGCCCACGATCCCAGACACTGAAGATGAAATAAGTCCAGACAAAGTAATACTTTGCTTGGAACAAAAACTCATCTCAGAAGAGGATCTG

CRBN (318-426aa) plasmid

ATGGCCGGCGAAGGAGATCAGCAGGACGCTGCGCACAACATGGGCAACCACCTGCCGCTCCTGCCTGCAGAGAGTGAGGAAGAAGATGAAATGGAAGTTGAAGACCAGGATAGTAAAGAAGCCAAAAAACCAAACATCATAAATTTTGACACCAGTCTGCCGACATCACATACATACCTAGGTGCTGATATGGAAGAATTTCATGGCAGGACTTTGCACGATGACGACAGCTGTCAGGTGATTCCAGTTCTTCCACAAGTGATGATGATCCTGATTCCCGGACAGACATTACCTCTTCAGCTTTTTCACCCTCAAGAAGTCAGTATGGTGCGGAATTTAATTCAGAAAGATAGAACCTTTGCTGTTCTTGCATACAGCAATGTACAGGAAAGGGAAGCACAGTTTGGAACAACAGCAGAGATATATGCCTATCGAGAAGAACAGGATTTTGGAATTGAGATAGTGAAAGTGAAAGCAATTGGAAGACAAAGGTTCAAAGTCCTTGAGCTAAGAACACAGTCAGATGGAATCCAGCAAGCTAAAGTGCAAATTCTTCCCGAATGTGTGTTGCCTTCAACCATGTCTGCAGTTCAATTAGAATCCCTCAATAAGTGCCAGATATTTCCTTCAAAACCTGTCTCAAGAGAAGACCAATGTTCATATAAATGGTGGCAGAAATACCAGAAGAGAAAGTTTCATTGTGCAAATCTAACTTCATGGCCTCGCTGGCTGTATTCCTTATATGATGCTGAGACCTTAATGGACAGAATCAAGAAACAGCTACGTGAATGGGATGAAAATCTAAAAGATGATTCTCTTCCTTCAAATCCAATAGATTTTTCTTACAGAGTAGCTGCTTGTCTTCCTATTGATGATGTATTGAGAATTCAGCTCCTTAAAATTGGCAGTGCTATCCAGCGACTTCGCTGTGAATTAGACATTATGAATAAAGACACTGAAGATGAAATAAGTCCAGACAAAGTAATACTTTGCTTGGAACAAAAACTCATCTCAGAAGAGGATCTG

HA-Ub plasmid

atgtacccatacgatgttccggattacgctagcggatccatgcagatcttcgtgaaaacccttaccggcaagaccatcacccttgaggtggagcccagtgacaccatcgaaaatgtgaaggccaagatccaggataaggaaggcattccccccgaccagcagaggctcatctttgcaggcaagcagctggaagatggccgtactctttctgactacaacatccagaaggagtcgaccctgcacctggtcctgcgtctgagaggtggttag

HIF1A plasmid

ATGGAGGGCGCCGGCGGCGCGAACGACAAGAAAAAGATAAGTTCTGAACGTCGAAAAGAAAAGTCTCGAGATGCAGCCAGATCTCGGCGAAGTAAAGAATCTGAAGTTTTTTATGAGCTTGCTCATCAGTTGCCACTTCCACATAATGTGAGTTCGCATCTTGATAAGGCCTCTGTGATGAGGCTTACCATCAGCTATTTGCGTGTGAGGAAACTTCTGGATGCTGGTGATTTGGATATTGAAGATGACATGAAAGCACAGATGAATTGCTTTTATTTGAAAGCCTTGGATGGTTTTGTTATGGTTCTCACAGATGATGGTGACATGATTTACATTTCTGATAATGTGAACAAATACATGGGATTAACTCAGTTTGAACTAACTGGACACAGTGTGTTTGATTTTACTCATCCATGTGACCATGAGGAAATGAGAGAAATGCTTACACACAGAAATGGCCTTGTGAAAAAGGGTAAAGAACAAAACACACAGCGAAGCTTTTTTCTCAGAATGAAGTGTACCCTAACTAGCCGAGGAAGAACTATGAACATAAAGTCTGCAACATGGAAGGTATTGCACTGCACAGGCCACATTCACGTATATGATACCAACAGTAACCAACCTCAGTGTGGGTATAAGAAACCACCTATGACCTGCTTGGTGCTGATTTGTGAACCCATTCCTCACCCATCAAATATTGAAATTCCTTTAGATAGCAAGACTTTCCTCAGTCGACACAGCCTGGATATGAAATTTTCTTATTGTGATGAAAGAATTACCGAATTGATGGGATATGAGCCAGAAGAACTTTTAGGCCGCTCAATTTATGAATATTATCATGCTTTGGACTCTGATCATCTGACCAAAACTCATCATGATATGTTTACTAAAGGACAAGTCACCACAGGACAGTACAGGATGCTTGCCAAAAGAGGTGGATATGTCTGGGTTGAAACTCAAGCAACTGTCATATATAACACCAAGAATTCTCAACCACAGTGCATTGTATGTGTGAATTACGTTGTGAGTGGTATTATTCAGCACGACTTGATTTTCTCCCTTCAACAAACAGAATGTGTCCTTAAACCGGTTGAATCTTCAGATATGAAAATGACTCAGCTATTCACCAAAGTTGAATCAGAAGATACAAGTAGCCTCTTTGACAAACTTAAGAAGGAACCTGATGCTTTAACTTTGCTGGCCCCAGCCGCTGGAGACACAATCATATCTTTAGATTTTGGCAGCAACGACACAGAAACTGATGACCAGCAACTTGAGGAAGTACCATTATATAATGATGTAATGCTCCCCTCACCCAACGAAAAATTACAGAATATAAATTTGGCAATGTCTCCATTACCCACCGCTGAAACGCCAAAGCCACTTCGAAGTAGTGCTGACCCTGCACTCAATCAAGAAGTTGCATTAAAATTAGAACCAAATCCAGAGTCACTGGAACTTTCTTTTACCATGCCCCAGATTCAGGATCAGACACCTAGTCCTTCCGATGGAAGCACTAGACAAAGTTCACCTGAGCCTAATAGTCCCAGTGAATATTGTTTTTATGTGGATAGTGATATGGTCAATGAATTCAAGTTGGAATTGGTAGAAAAACTTTTTGCTGAAGACACAGAAGCAAAGAACCCATTTTCTACTCAGGACACAGATTTAGACTTGGAGATGTTAGCTCCCTATATCCCAATGGATGATGACTTCCAGTTACGTTCCTTCGATCAGTTGTCACCATTAGAAAGCAGTTCCGCAAGCCCTGAAAGCGCAAGTCCTCAAAGCACAGTTACAGTATTCCAGCAGACTCAAATACAAGAACCTACTGCTAATGCCACCACTACCACTGCCACCACTGATGAATTAAAAACAGTGACAAAAGACCGTATGGAAGACATTAAAATATTGATTGCATCTCCATCTCCTACCCACATACATAAAGAAACTACTAGTGCCACATCATCACCATATAGAGATACTCAAAGTCGGACAGCCTCACCAAACAGAGCAGGAAAAGGAGTCATAGAACAGACAGAAAAATCTCATCCAAGAAGCCCTAACGTGTTATCTGTCGCTTTGAGTCAAAGAACTACAGTTCCTGAGGAAGAACTAAATCCAAAGATACTAGCTTTGCAGAATGCTCAGAGAAAGCGAAAAATGGAACATGATGGTTCACTTTTTCAAGCAGTAGGAATTGGAACATTATTACAGCAGCCAGACGATCATGCAGCTACTACATCACTTTCTTGGAAACGTGTAAAAGGATGCAAATCTAGTGAACAGAATGGAATGGAGCAAAAGACAATTATTTTAATACCCTCTGATTTAGCATGTAGACTGCTGGGGCAATCAATGGATGAAAGTGGATTACCACAGCTGACCAGTTATGATTGTGAAGTTAATGCTCCTATACAAGGCAGCAGAAACCTACTGCAGGGTGAAGAATTACTCAGAGCTTTGGATCAAGTTAACGGGGGTGGAGGCTCTGAGCAGAAACTCATCTCAGAAGAGGATCTGTAA

Green: Myc
