# Supplementary material for: Thalidomide targets EGFL6 to inhibit EGFL6/PAX6 axis-driven angiogenesis in small bowel vascular malformation
Source: Cell Mol Life Sci. 2020 Feb 1;77(24):5207–21. doi: 10.1007/s00018-020-03465-3 (PMC7671996; doi:10.1007/s00018-020-03465-3)
Supplement: Supplementary file 3 — Supplementary material 3 (DOCX 899 kb) [file 18_2020_3465_MOESM3_ESM.docx]

Supplementary Figure 1


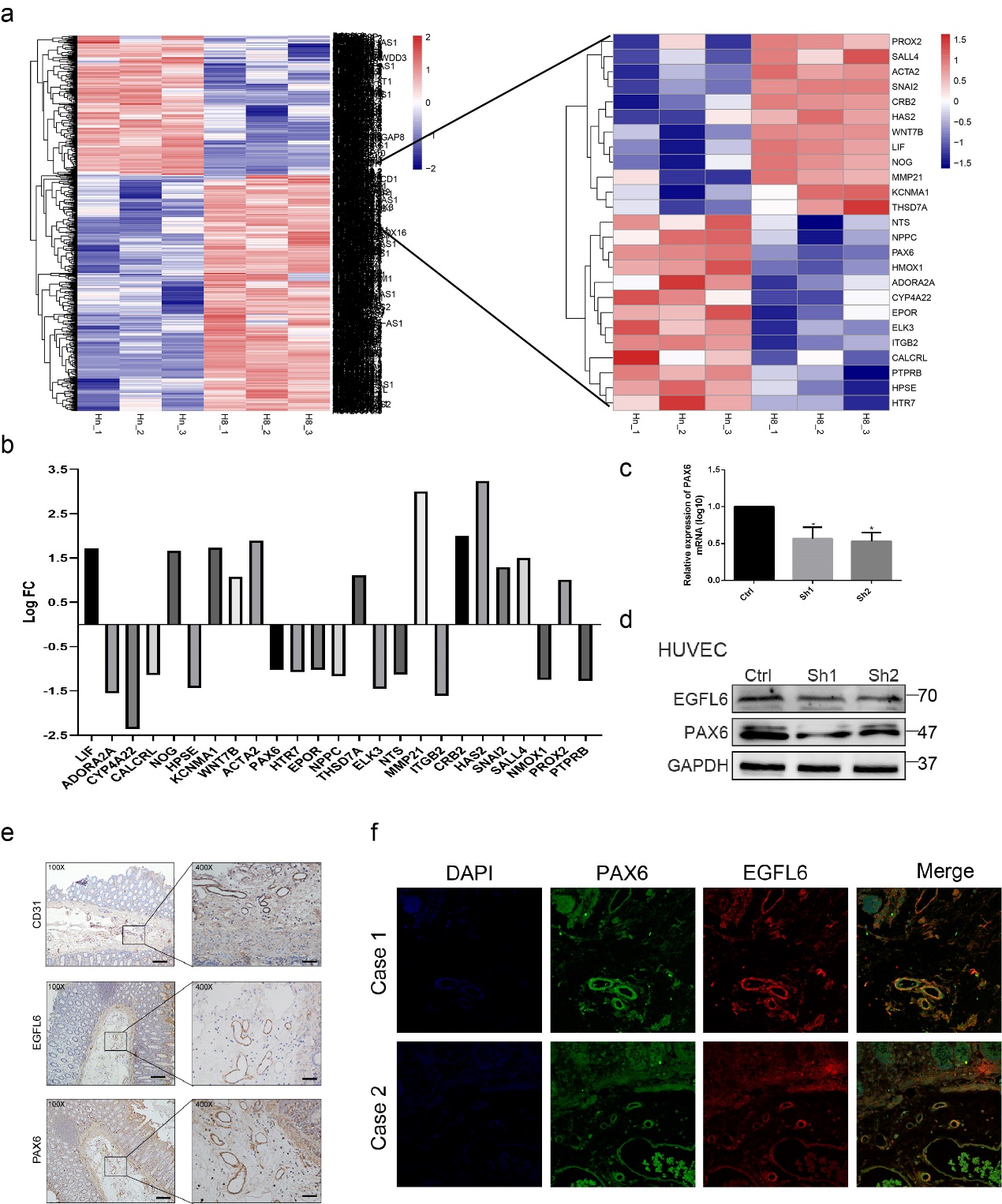


**Supplementary Figure 1: PAX6 was identified as the downstream factor in EGFL6-mediated angiogenesis. a** Differential gene expression in EGFL6 knockdown group and control group was identified by RNA sequence (left), and we analyzed the differential gene expression associated with angiogenesis (right). **b** The value of log2 fold-change of these genes expression. **c-d** RT-PCR and western blot were performed to test the results of RNA sequence after transfection of EGFL6 siRNA. **e** Representative pictures of SBVM samples stained with CD31, EGFL6 and PAX6. Scale bars: left, 200um; right, 50um. **f** Immunofluorescence Assay were performed to detect the colocalization between EGFL6 and PAX6 in SBVM tissues.

Supplementary Figure 2


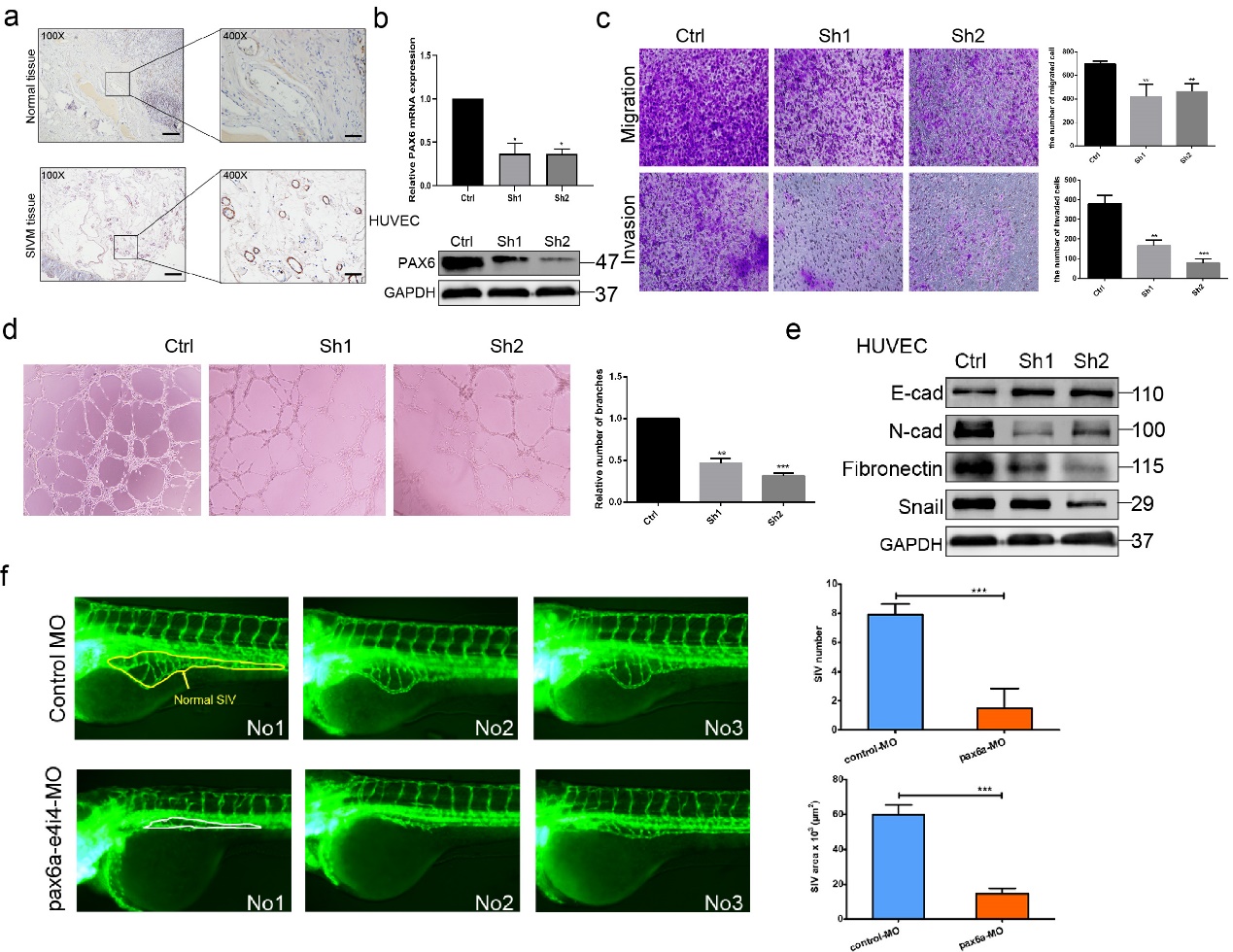


**Supplementary Figure 2：PAX6 promotes angiogenesis in vitro and in vivo. a** The results of IHC against PAX6 in SBVM samples and normal tissues. Scale bars: 100um; **b** cells migration and invasion assay with HUVEC transfected with siNC and PAX6 siRNA, represented image was shown on the left and Quantitative data are expressed as mean ± SEM , **P < 0.01, ***P < 0.001. **c** tuber formation assay with HUVEC transfected with siNC and PAX6 siRNA, represented image was shown on the left and result was shown by bar chart, **P < 0.01, ***P < 0.001. **d** The expression of molecules correlated with cell invasion was analyzed by western blot. E Representative fluorescent images of control-MO or embryos treated with *pax6a*-MO at 3 days post-fertilization (dpf). In control embryos, subintestinal vein vessels (SIVs) developed as a smooth basket-like structure over the yolk at 3-dpf ( yellow dashed lines). In contrast, embryos injected with *pax6a*-MO resulted in specific defects in subintestinal vein vessels (SIVs) formation (white dashed lines), quantification of the area and number of SIV shows significantly decreased in *pax6a* morphants. ***P < 0.0001(n =10; Student’s t test.). Data represent the average of 3 independent experiments.
