# Supplementary figures and images for: Thalidomide targets EGFL6 to inhibit EGFL6/PAX6 axis-driven angiogenesis in small bowel vascular malformation
Source: Cell Mol Life Sci. 2020 Feb 1;77(24):5207–21. doi: 10.1007/s00018-020-03465-3 (PMC7671996; doi:10.1007/s00018-020-03465-3)

HEK 293T cells certificate


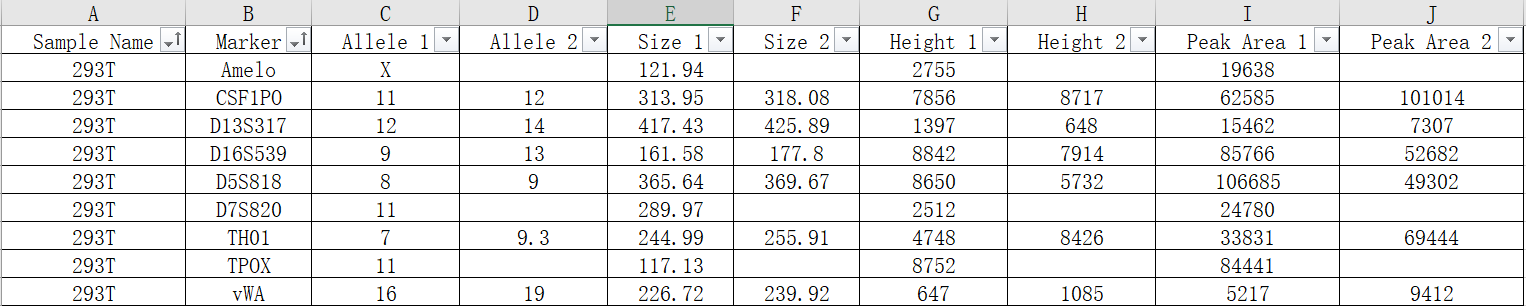


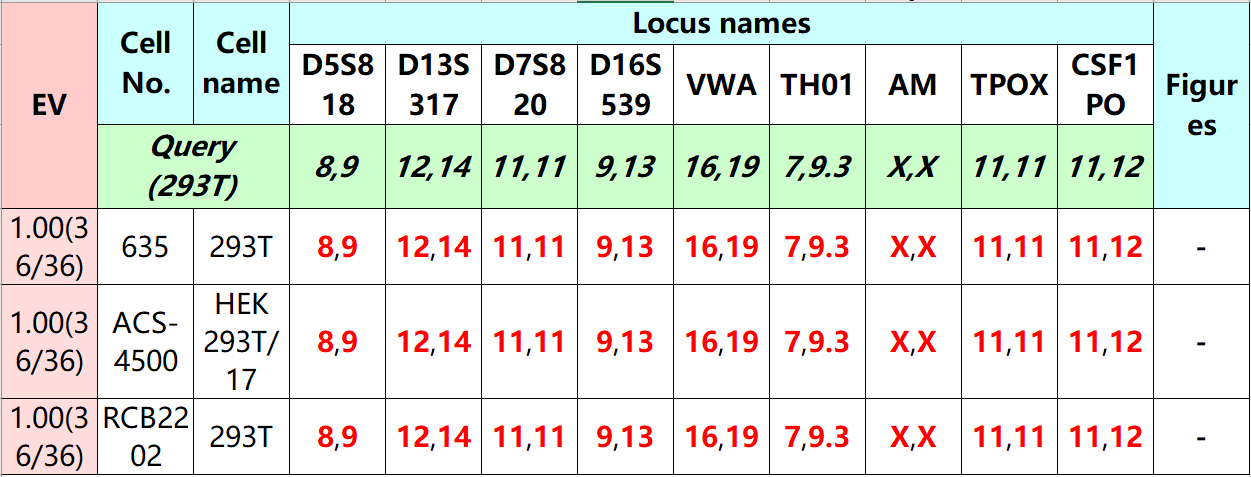


HUVEC certificate


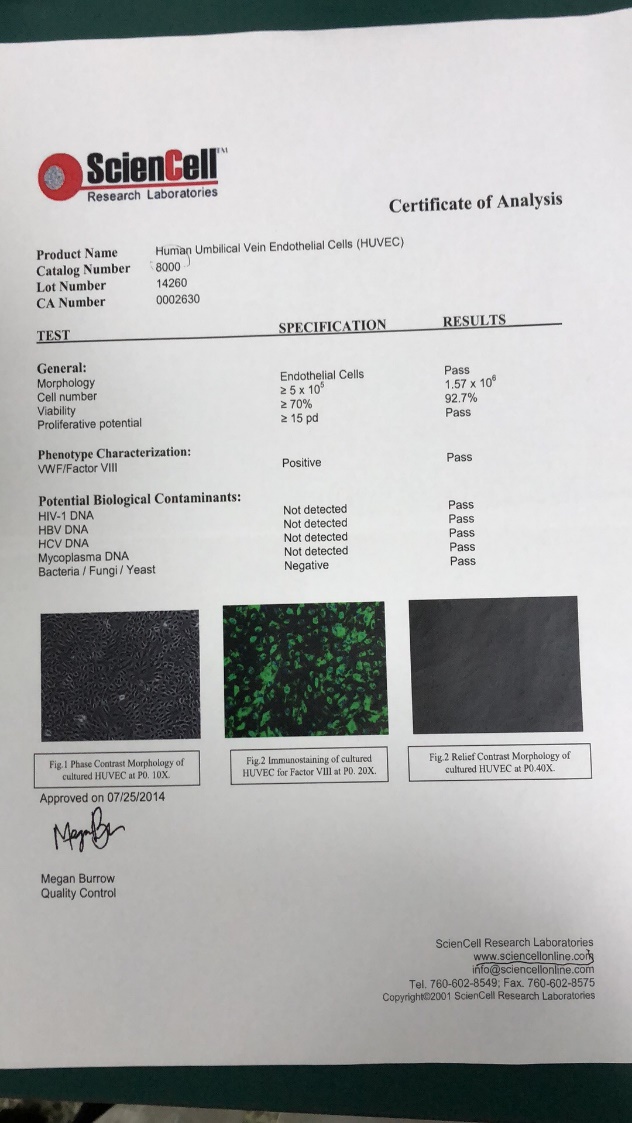

Supplement: Supplementary file 4 — Supplementary material 4 (DOCX 288 kb) [file 18_2020_3465_MOESM4_ESM.docx]
